# Heterozygous *NFKB1* variant causes inflammatory dysregulation shaped by broader genetic context in common variable immunodeficiency

**DOI:** 10.1172/jci.insight.198703

**Published:** 2026-03-23

**Authors:** Kevin M. Hayes, Kai Boldt, Peter J. Schnorr, Pushpinder Bawa, Miranda L. Abyazi, Matthew S. Ware, Gavin Gyimesi, Marianne James, Huaibin M. Ko, Charlotte Cunningham-Rundles, Joseph P. Mizgerd, Gustavo Mostoslavsky, Darrell N. Kotton, Paul J. Maglione

**Affiliations:** 1 Pulmonary Center and Section of Pulmonary, Allergy, Sleep and Critical Care, Department of Medicine, Boston University Chobanian & Avedisian School of Medicine, Boston, Massachusetts, USA.; 2 Center for Regenerative Medicine, Boston University and Boston Medical Center, Boston, Massachusetts, USA.; 3 Department of Biological Sciences, Fordham University, Bronx, New York, USA.; 4 Department of Pathology & Cell Biology, Columbia University Irving Medical Center, New York, New York, USA.; 5 Division of Clinical Immunology, Departments of Medicine and Pediatrics, Icahn School of Medicine at Mount Sinai, New York, New York, USA.

**Keywords:** Immunology, Inflammation, Innate immunity, NF-kappaB

## Abstract

Common variable immunodeficiency (CVID) is the most prevalent symptomatic primary antibody deficiency. For unclear reasons, inflammatory complications, like gastrointestinal (GI) disease, occur in ~50% of CVID cases, worsening morbidity and mortality. *NFKB1* variants are among the most frequent genetic variants in CVID. While effect of *NFKB1* variants is not well understood, we previously found frameshift heterozygous *NFKB1* variants to increase cytokines, monocytes, and inflammatory complications in CVID. In this report, we used induced pluripotent stem cell–derived (iPSC-derived) monocytes (iMONOs) with CRISPR/Cas9-mediated gene editing to study a heterozygous *NFKB1* frameshift found in a patient with CVID with severe GI disease. The heterozygous *NFKB1* variant similarly reduced NFKB1 protein in CVID patient– and healthy donor–derived iMONOs, but elevated LPS-induced IL-1β release and expression of inflammatory genes, including *IL1B*, *IL6*, *TNF*, and neutrophil chemoattractants, only in CVID patient iMONOs. CVID patient iMONOs also had elevations of IL-12, CCL4, and CCL12 unaffected by presence or absence of the *NFKB1* variant. TNF antagonism improved the patient’s GI disease, diminishing neutrophilic gastritis, circulating neutrophils, and the neutrophil chemoattractant CXCL1 in the blood. While the biology remains complex, our approach found heterozygous *NFKB1* variant–induced inflammatory changes intensified in CVID iMONOs, corresponding with clinical response to TNF antagonism.

## Abstract 2

This study uses induced pluripotent stem cells and targeted gene editing to understand how a frameshift *NFKB1* variant promotes severe disease in common variable immunodeficiency.

## Graphical abstract

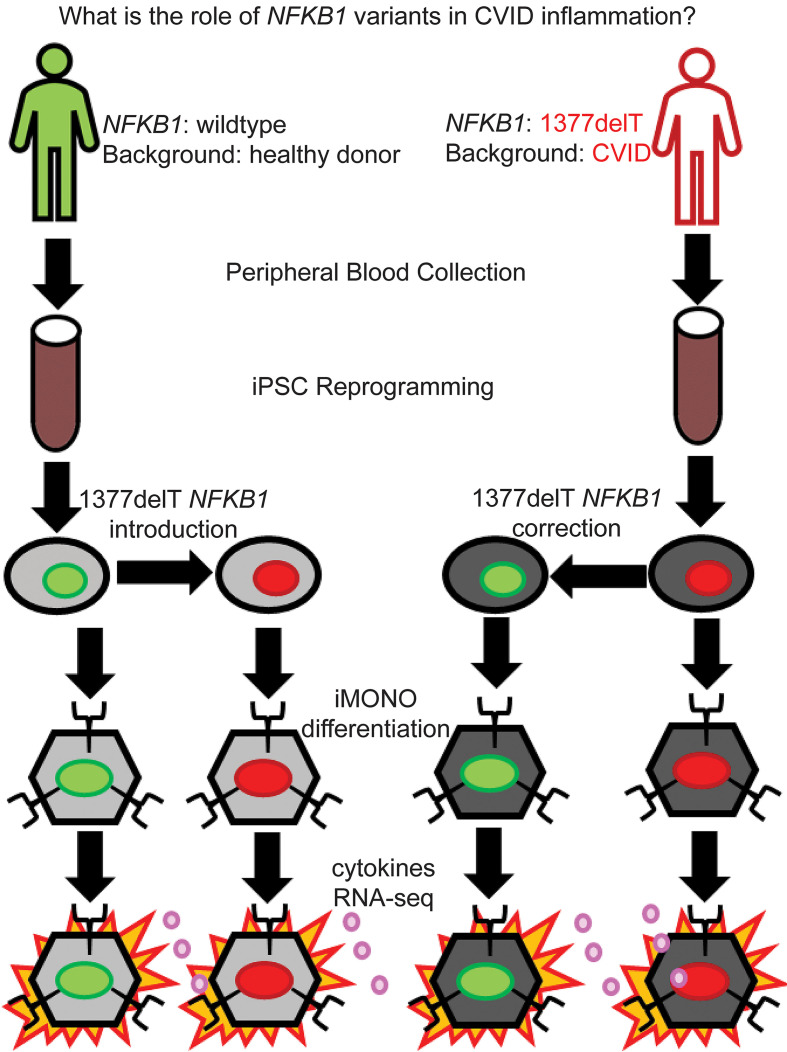

## Introduction

Common variable immunodeficiency (CVID) is the most clinically significant of the predominantly antibody deficiency disorders, which collectively are the most numerous inborn errors of immunity overall (1). CVID affects an estimated 1:25,000, and is defined by reduced levels of IgG, IgA, and/or IgM with impaired specific antibody response (2, 3). CVID clinical course varies, with all having increasing susceptibility to infection but about half also developing noninfectious complications, such as inflammatory gastrointestinal (GI) disease, for unclear reasons (4). As noninfectious complications significantly worsen morbidity and mortality of CVID despite currently available treatments, better understanding of underlying pathogenesis is a critical unmet need in CVID that could provide much-needed advancement of therapy (5–8).

Heterozygous *NFKB1* variants are among the most frequent CVID-associated genetic findings (9). *NFKB1* encodes both the full-length protein p105 and its truncated cleavage product p50, proteins that function in both activation and regulation of canonical NF-κB signaling (10, 11). Notably, *NFKB1* variants are more common in those with inflammatory disease than symptomatic antibody deficiency, indicating that they may promote inflammatory disease in CVID (12). Elevated cytokine production was reported in a patient with CVID with an *NFKB1* variant and severe GI disease, and a case series study found *NFKB1* variants associated with inflammasome activation and other inflammatory dysregulation, though the participants in that report did not satisfy CVID diagnostic criteria (13, 14). Recently, heterozygous *NFKB1* variants, particularly those that are frameshift or nonsense, were associated with increased inflammatory complications in a CVID cohort, together with elevated circulating monocytes and increased plasma cytokines (15). In vitro overexpression systems have shown that CVID-associated *NFKB1* variants cause protein haploinsufficiency (16, 17). However, in vitro overexpression may not model the variant heterozygosity occurring in patients or provide insight into downstream inflammatory effects that may shape CVID manifestations.

Induced pluripotent stem cells (iPSCs) can be generated from peripheral blood, serving as a self-renewing reservoir to derive cells with donor-specific genetics (18, 19). CRISPR/Cas9-mediated gene editing can introduce or correct genetic variants in iPSCs, which then can be differentiated into disease-relevant cells of interest for further investigation (20–22). An iPSC-based approach offers several advantages over prior studies. First, it can isolate effect of heterozygous *NFKB1* variants from other genetic differences through use of targeted gene editing of alternate genetic backgrounds. Second, it achieves heterozygous, endogenous expression as occurs in patients. Third, it can help distinguish genetic effects from epigenetic and chronic inflammatory changes that obscure the basis of an inflammatory phenotype in primary cells taken from patients with active disease because iPSCs have reduced, though not necessarily absent, epigenetic changes (23). Lastly, we are able to isolate a specific cell type to study in the context of CVID inflammatory disease.

Monocyte activation and expansion are features of patients with CVID with inflammatory disease complications, including those with heterozygous *NFKB1* variants (15, 24–27). Monocytes are also a significant contributor to cytokine elevation in CVID; phospho-STAT1^+^ monocytes are a primary source of B cell activating factor elevation found increased in these patients, possibly downstream of the elevated IFN-γ and *STAT1* expression that define these patients (28). Given this precedent for monocyte dysregulation in CVID inflammatory complications, we used iPSC-derived monocytes (iMONOs) as our cellular model to elucidate effect of a heterozygous *NFKB1* variant.

Using iMONOs together with gene editing, we aimed to elucidate the effect of a heterozygous frameshift *NFKB1* variant found in a patient with CVID with a severe inflammatory course, specifically a form of refractory GI disease that had profound clinical response to TNF antagonism. To our knowledge, this is the first application of iPSCs in the study of CVID. We explored effects of the heterozygous *NFKB1* variant in different genetic backgrounds, including the effect of gene correction upon immune dysregulation. This approach made it possible to delineate effect of a heterozygous frameshift *NFKB1* variant from the other genetic factors in a patient with CVID with severe inflammatory disease and provide rationale for the therapeutic response to TNF antagonism. Our results paint a more complex picture of the relationship between *NFKB1* variants and inflammatory disease in CVID than previously thought, exposing influence of the broader genetic background in which the variant is present and demonstrating therapeutic potential of targeting NF-κB–related immune dysregulation.

## Results

### Severe and refractory GI disease in a patient with CVID with 1377delT NFKB1 variant.

**Figure 1:**
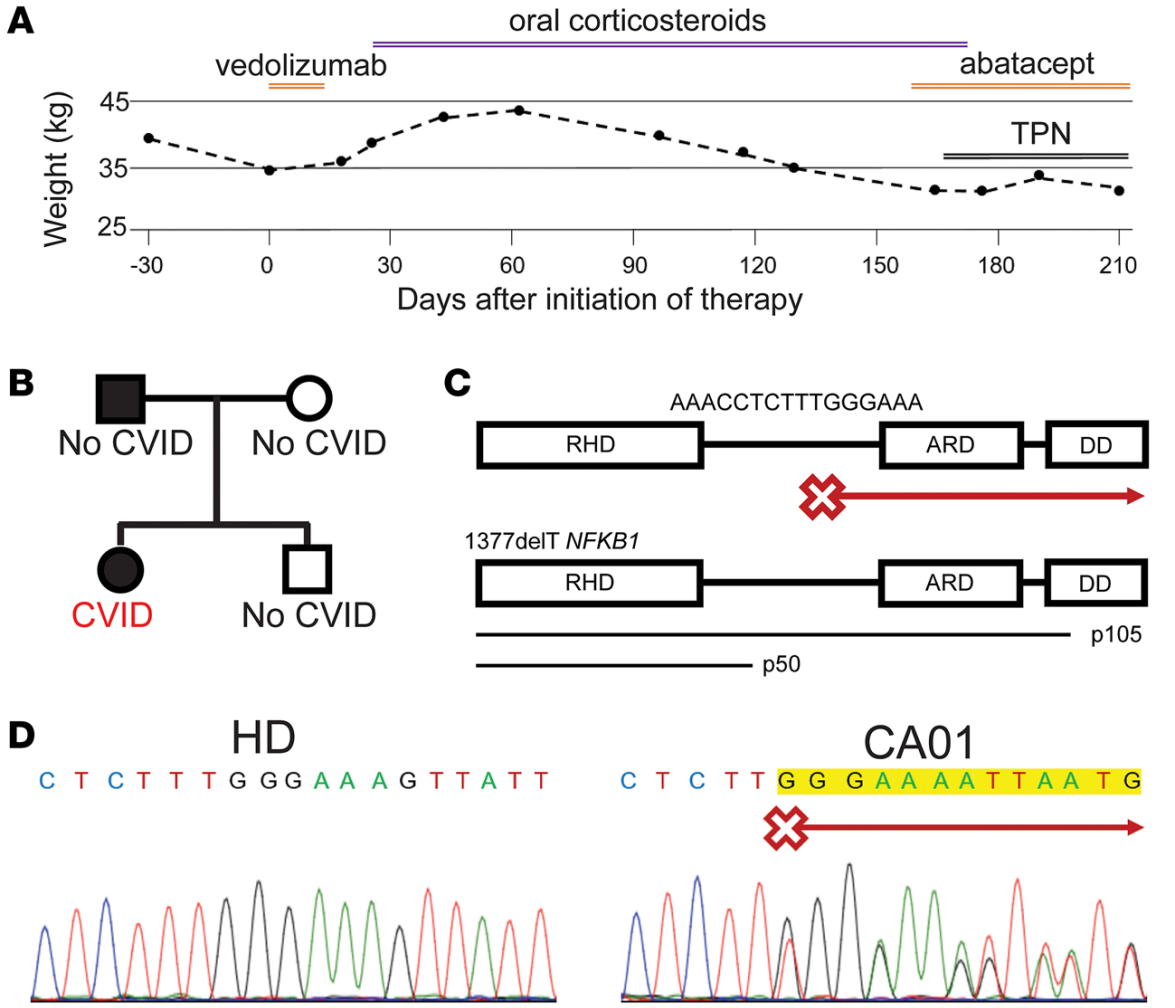
Severe and refractory GI disease in a patient with CVID with heterozygous 1377delT *NFKB1* variant (patient CA01). (**A**) Difficulty to maintain healthy weight persists despite several therapeutic approaches. TPN, total peripheral nutrition. (**B**) Family pedigree for heterozygous 1377delT *NFKB1* variant. (**C**) Schematic illustration of *NFKB1*, p50, and p105 coding regions as denoted. Location of 1377delT variant as indicated in red. RHD, Rel homology domain; ARD, ankyrin repeat domain; DD, death domain. (**D**) *NFKB1* sequence from PBMCs by Sanger sequencing for healthy donor (HD) on left and CA01 on right. 1377delT frameshift denoted in red.

The focus of our report is a 26-year-old female patient (referred to as patient CA01) who was diagnosed with CVID at age 8 years and was receiving immunoglobulin replacement therapy. While her diagnostic IgG value was not available, her current IgA and IgM values were both below the level of detection for the clinical laboratory. Peripheral leukocyte counts demonstrated elevated neutrophils at 11,500 cells/μL (reference range, 1800–7000 cells /μL), monocyte count of 900 cells /μL (200–900 cells /μL), CD4^+^ T cell count of 1129 cells /μL (430–1185 cells /μL), CD8^+^ T cells of 552 cells /μL (180–865 cells /μL), and NK cells of 64 cells /μL (48–450 cells /μL) and no detectable B cells. Clinical history is notable for prior GI infections with *Campylobacter jejuni*, giardia, and recurrent *Clostridium difficile* infections as well as bronchiectasis, gastritis, and enteropathy. CA01 had severe chronic GI disease with malabsorption and extreme difficulty maintaining a healthy weight. As intraepithelial lymphocytosis of the small bowel was observed on biopsy, enteropathy was presumed to be the etiology of the patient’s failure to thrive, and several immunomodulatory treatments were tried. Vedolizumab, an integrin α4β7 antagonist that blocks GI lymphocyte recruitment, was stopped after a brief course due to drug-induced liver injury. Additionally, oral steroids (prednisone and budesonide) and abatacept, CTLA-4 Ig, which acts as checkpoint repressor of T cells, failed to achieve sustained improvement (Figure 1A). The patient continued to worsen and required total peripheral nutrition (TPN) due to this severe and refractory GI disease.

Whole exome sequencing was performed, identifying a heterozygous 1377delT variant in *NFKB1* not found in genome databases. Her father also had the 1377delT heterozygous *NFKB1* variant and lives in Puerto Rico with limited contact with CA01, sharing 1 other child with the patient’s mother, who does not carry the variant (Figure 1B). The father has no known immunodeficiency, though he has had unspecified GI disease requiring medical care. The 1377delT variant results from a deleted thymidine between the p50 and p105 coding regions of *NKFB1*, resulting in disruption of the downstream reading frame (Figure 1C). This was confirmed via Sanger sequencing of amplicons targeting *NKFB1* Exon 14 from gDNA of CA01 peripheral blood mononuclear cells (PBMCs) (Figure 1D). The chromatograms of this sequence introduce mixed, equivalent peaks downstream of the affected thymine, as would be expected of a heterozygous frameshift variant.

### Elevation of NF-κB–driven cytokines, including TNF, and clinical response to TNF antagonism in patient with CVID with heterozygous 1377delT NFKB1 variant.

**Figure 2:**
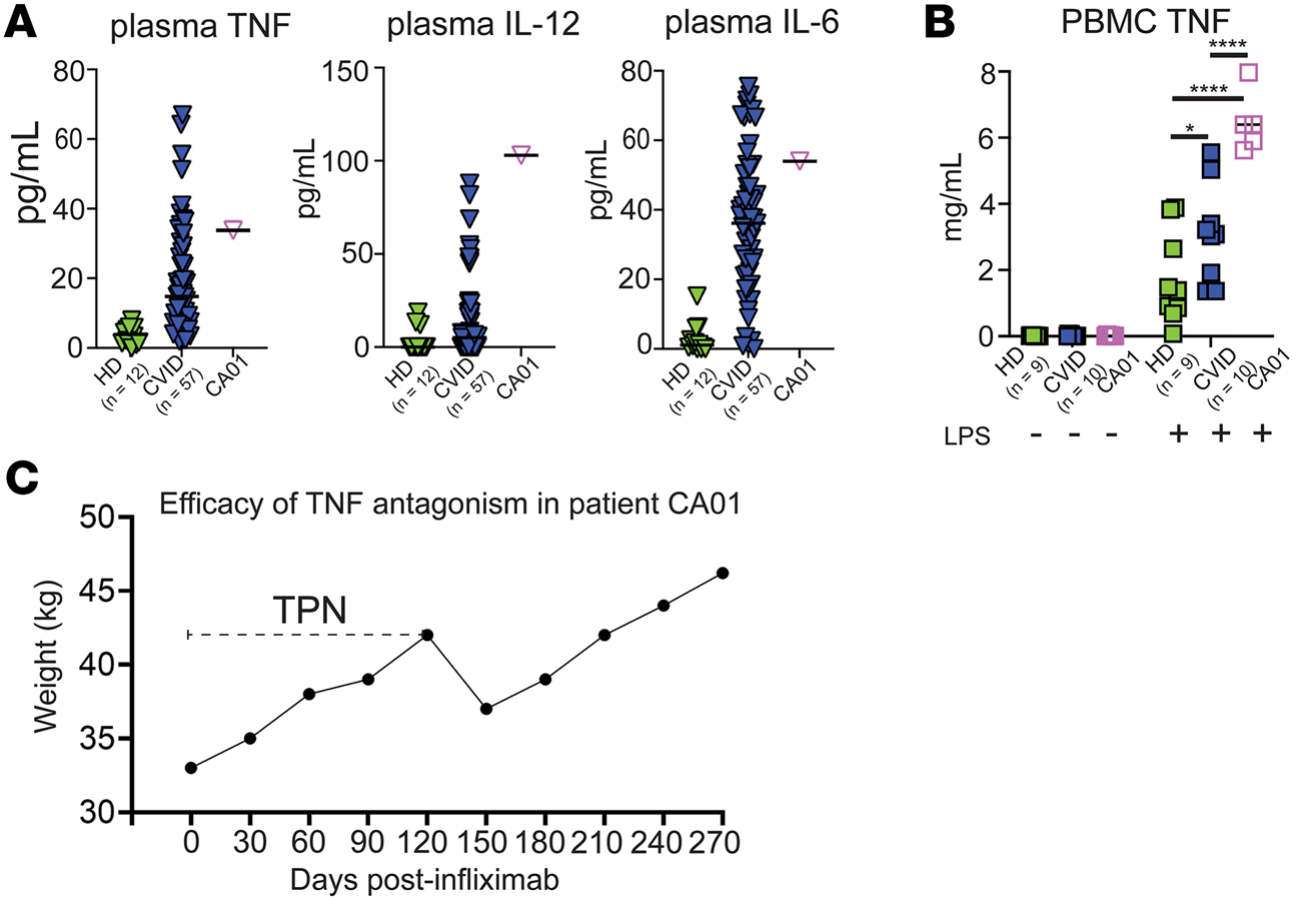
Elevation of NF-κB–driven cytokines, including TNF, and clinical response to TNF antagonism in a patient with CVID with heterozygous 1377delT *NFKB1* variant. (**A**) Levels of TNF, IL-12, and IL-6 measured in plasma by ELISA. *P* value calculated by 1-way ANOVA and Kruskal-Wallis test for multiple comparisons. (**B**) TNF levels in 18 hours PBMC culture media with or without 5 ng/mL LPS measured by ELISA. Results from 9 HD donors and 10 CVID donors, and 5 monthly blood draws from CA01. *P* value calculated by 2-way ANOVA with Holm-Šídák test for multiple comparisons. (**C**) Effect of infliximab on CA01 weight over time. PBMC, peripheral blood mononuclear cell; TPN, total peripheral nutrition. *P < 0.05, ****P < 0.0001.

CA01, the patient with CVID with a heterozygous 1377delT *NFKB1* variant, had elevated levels of the NF-κB–driven cytokines IL-6, IL-12, and TNF in plasma, above the seventy-fifth percentile for each of these cytokines in our CVID cohort and above levels in healthy donors (HD) (Figure 2A). We then tested the effect of LPS, a TLR4 agonist that is a potent inducer of these cytokines via NF-κB, on PBMC cultures, knowing LPS is also elevated in circulation of patients with CVID (29–31). Addition of LPS resulted in elevation of TNF release by CA01 PBMCs (5 replicates from 5 distinct blood draws over the course of 5 months) (Figure 2B). Difference was significant compared with PBMCs from 10 different patients with CVID and 9 different HD, only for LPS-stimulated cultures. Due to TPN-dependent GI disease and evidence of cytokine dysregulation, CA01 was treated with the TNF inhibitor infliximab. In the months while on this therapy, CA01 regained weight, no longer required TPN, and ultimately regained an excellent quality of life, returning to full-time work (Figure 2C).

### Establishment of an isogenic iMONO model of NFKB1-variant immune dysregulation.

We employed an iPSC-based approach that used CRISPR/Cas9-mediated gene editing to prepare multiple syngeneic lines with versus without the *NFKB1* variant in order to profile the effects of the variant while controlling for genetic backgrounds. Specifically, we engineered 2 paired clones from each independent genetic background: (a) iPSCs derived from the patient carrying the variant with parallel iPSCs corrected by editing, and (b) normal HD iPSCs with parallel HD iPSCs edited to introduce the variant. This approach is anticipated to remove the potential confounding factors of epigenetic alterations and chronic inflammatory changes that can complicate analysis of primary cells from a patient with CVID with active inflammatory disease, like CA01 (32).

We reprogrammed iPSCs from CA01 and used previously published HD iPSCs (clone BU3) (19). Next, we repaired the 1377delT *NKFB1* variant to WT in CA01 iPSCs using CRISPR/Cas9 gene editing. In parallel, we introduced the heterozygous 1377delT *NFKB1* variant in HD iPSCs by gene editing, testing sufficiency of this variant to affect observed phenotypes into other genetic backgrounds. All 4 iPSC lines (CA01-derived iPSCs with and without heterozygous 1377delT *NFKB1* corrected to WT, HD-derived iPSCs with and without 1377delT *NFKB1* heterozygous introduction) exhibited normal growth, morphologies, and karyotypes as well as expected *NFKB1* sequences, including 50% with the intended variant sequence as consistent with a heterozygous variant (Supplemental Figure 1; supplemental material available online with this article; https://doi.org/10.1172/jci.insight.198703DS1).

**Figure 3:**
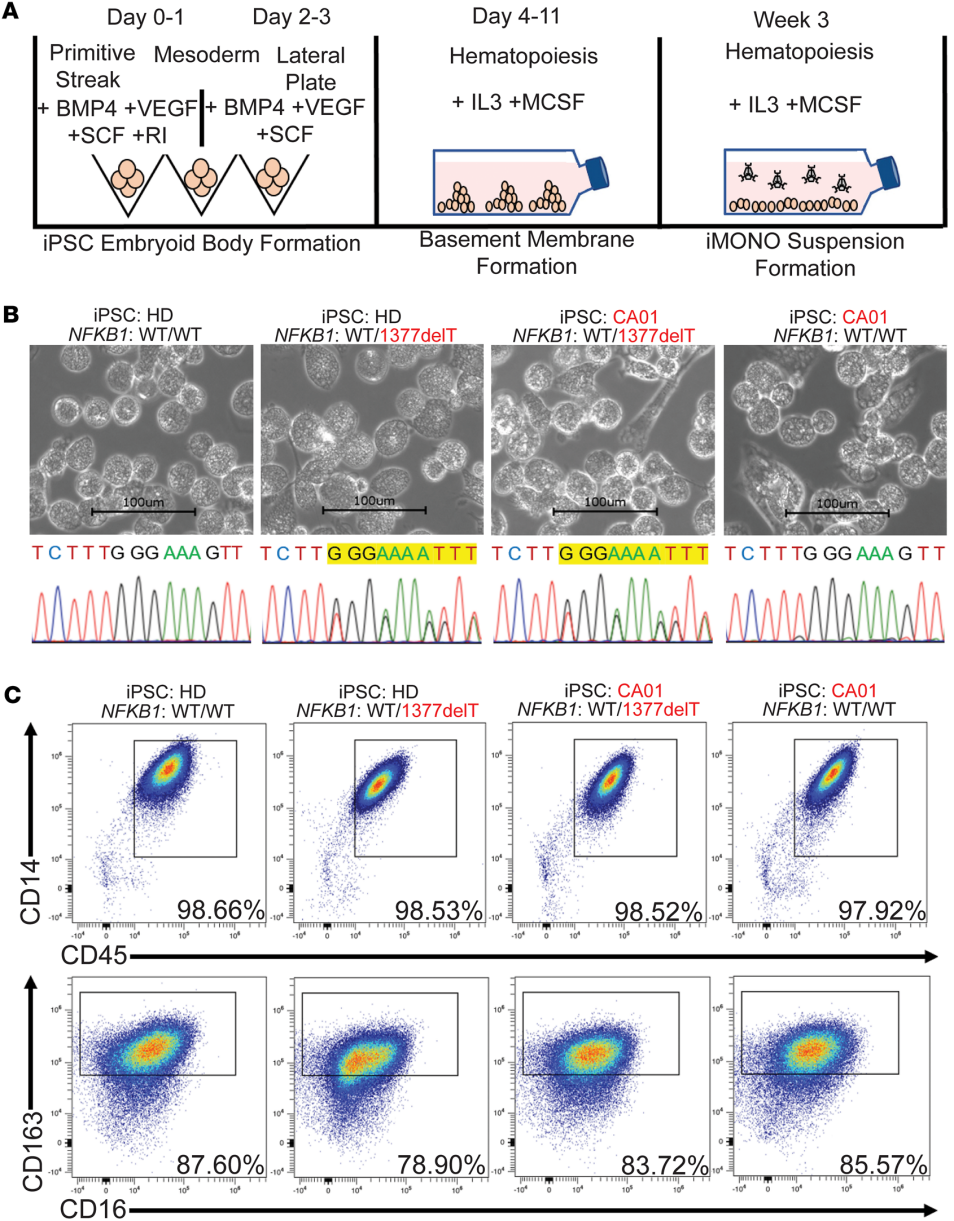
Establishment of an iPSC-derived monocyte (iMONO) model of *NFKB1*-variant immune dysregulation. (**A**) Schematic of protocol used to generate monocytes from iPSCs. (**B**) Morphology and DNA sequence in region of 1377delT *NFKB1* variant (highlighted in yellow) for each iPSC line used in this study. (**C**) Flow cytometry of CD45/CD14 and CD16/CD163 on iMONOs generated from each iPSC line used in this study. Data representative of more than 12 iMONO differentiations. Genetic background of iPSC line and presence of heterozygous *NFKB1* variant as denoted.

To generate monocytes from iPSCs, we used the method of directed differentiation, adapting the protocol of van Wilgenburg et al. to first differentiate each line into embryoid bodies (EB), which were patterned into lateral plate mesoderm using BMP4, VEGF, and SCF (33) (Figure 3A). After transfer of the resulting cells onto gelatin and treatment with M-CSF and IL-3, putative monocyte-like cells (iMONOs) emerged in suspension culture from their adherent precursors (Figure 3, A and B). We harvested putative iMONOs from each line for profiling, finding they retained the desired *NFKB1* genotypes and exhibited similar morphologies (Figure 3B). We monitored iMONO cell counts and viabilities by trypan blue staining and an automated cell counter across several weeks of differentiation. Variation in this data was not statistically significant between genetic backgrounds or *NFKB1* genotypes at any point. In addition, iMONOS across all lines produced similar populations of cells expressing monocyte marker CD14 together with hematopoietic marker, CD45 (>90% double positive) (Figure 3C). We did not find CD14 mean fluorescence intensity to differ between groups. CD45^+^CD14^+^ iMONOs also expressed similar levels of CD163 (monocyte/macrophage marker) and had low CD16 expression, consistent with the monocyte subset found elevated in blood of patients with CVID with inflammatory complications (24). Our gating strategy for iMONOs is outlined in Supplemental Figure 2.

### Heterozygous NFKB1 1377delT variant is necessary and sufficient to cause p105/p50 haploinsufficiency in iMONOs.

**Figure 4:**
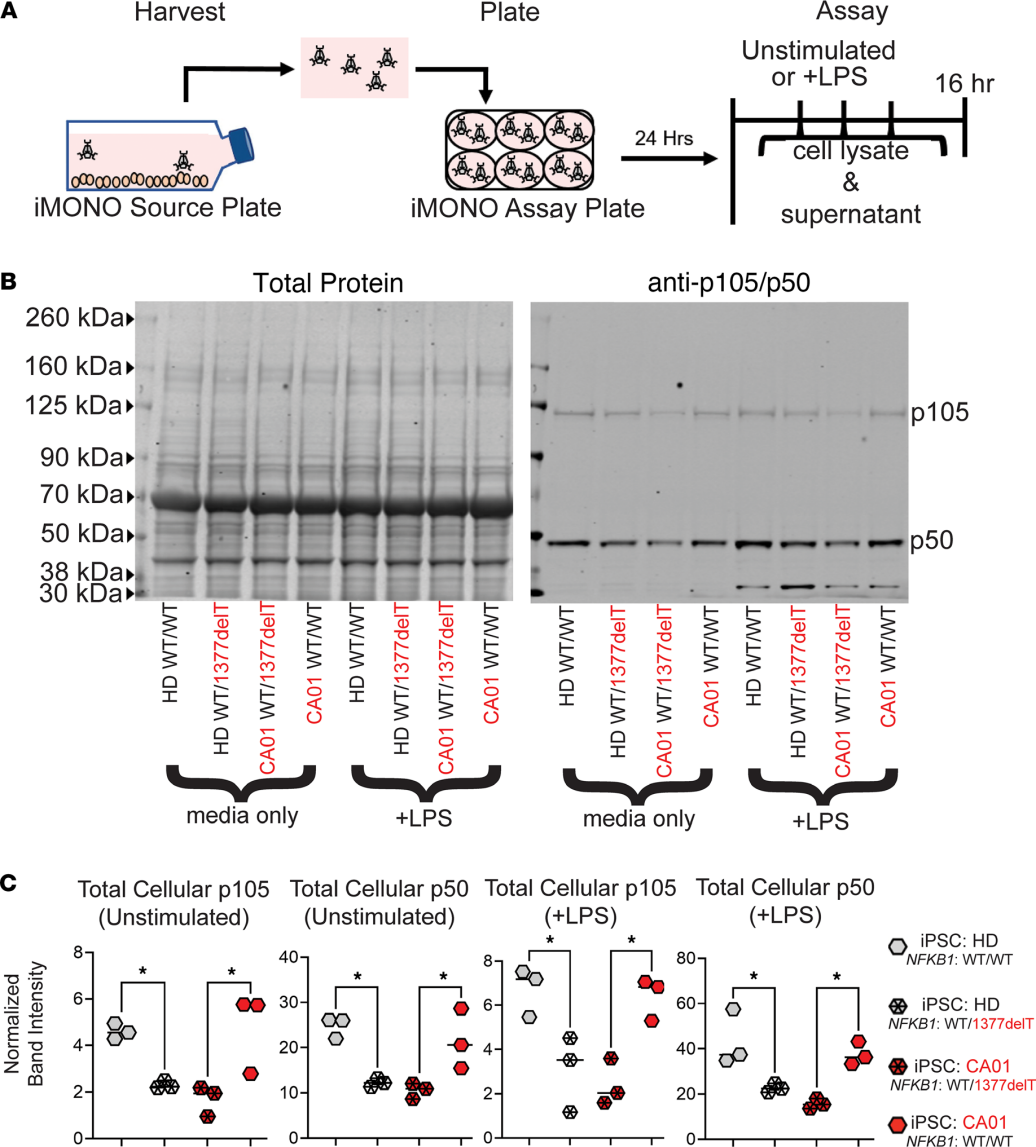
Heterozygous *NFKB1* 1377delT variant is necessary and sufficient to cause p105/p50 haploinsufficiency in iMONOs. (**A**) Schematic of protocol for iMONO assay. (**B**) Whole cell iMONO protein lysate (20 μg) analyzed for p105/p50 protein level by Western blot. Source of iPSC and present of *NFKB1* variant as indicated. (**C**) Densitometry analysis of Western blot bands from 3 blots generated from 3 independent experiments. *P* value was calculated by 1-way ANOVA with Holm-Šídák test for multiple comparisons. *P < 0.05.

We next measured levels of the *NFKB1*-encoded proteins, p105 and its processed form p50, by Western blot in iMONOs after culture with and without LPS (Figure 4A). Protein bands measured corresponded with the 105 kDA and 50 kDA molecular weights for p105 and p50, respectively (Figure 4B). Comparing band density normalized to total protein loaded, we observed that expression of heterozygous *NKFB1* 1377delT was necessary to reduce the NFKB1 protein by ~50% in the CVID patient–derived iMONOs and was sufficient to also reduce NFKB1 protein by ~50% in HD-derived iMONOs (Figure 4C). This effect was similar for both p50 and p105 and present under both unstimulated and LPS-stimulated conditions. In order to corroborate results from the iMONOs, we also examined PBMCs from the patient with CVID with the heterozygous 1377delT *NFKB1* variant along with 2 other patients with CVID with heterozygous *NFKB1* variants. We found reduced levels of p105 in the cytosolic fraction and p50 in the nuclear fraction both with and without LPS stimulation compared with HD (Supplemental Figure 3A). We also analyzed protein expression of the other NF-κB family members in iMONOs but found no significant differences under unstimulated or LPS stimulated conditions (Supplemental Figure 3B).

### iMONO cytokines are shaped by both heterozygous NFKB1 variant and broader CVID genetic background.

**Figure 5:**
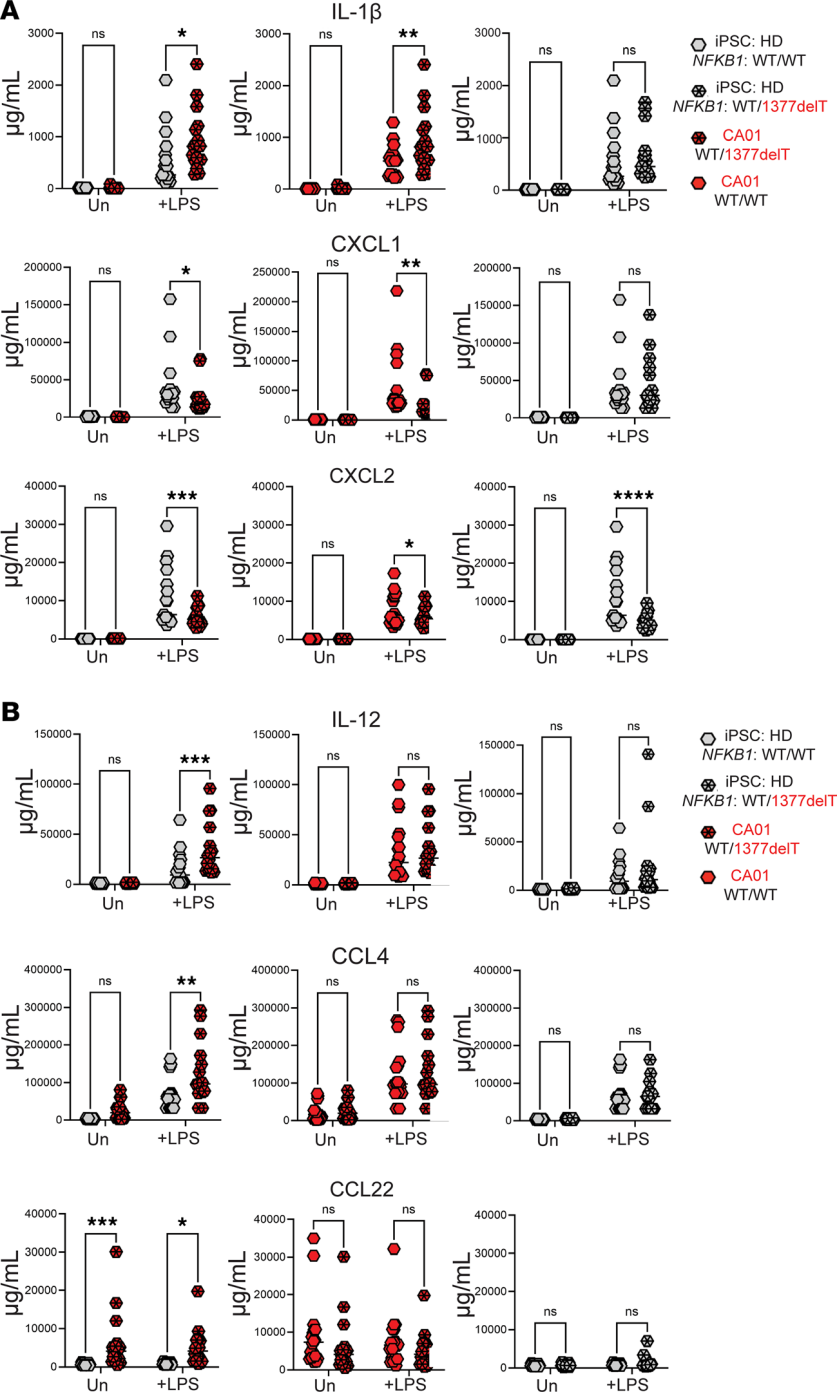
iMONO cytokines are shaped by both heterozygous *NFKB1* variant and broader genetic background. (**A**) IL-1β, CXCL1, and CXCL2 measured in supernatant of iMONO cultures with and without LPS. (**B**) IL-12, CCL4, and CCL22 measured in supernatant of iMONO cultures with and without LPS. Parental iPSC line (CA01 or HD) and presence of WT or 1377delT *NFKB1* for both alleles as noted. Each data point represents an experimental replicate. *P* value was calculated by 2-way ANOVA with Holm-Šídák test for multiple comparisons. *P < 0.05, **P < 0.01, ***P < 0.001, ****P < 0.0001.

To measure cytokine protein release from iMONOs we used the same culture conditions as with Western blot (Figure 4A). Using multiplex cytokine measurement, we found that IL-1β, CXCL1, and CXCL2 levels were significantly altered by the presence of the heterozygous 1377delT *NFKB1* variant in the CA01 background (Figure 5A). Presence of the heterozygous 1377delT *NFKB1* variant was necessary for IL-1β alteration in CA01 iMONOs but not sufficient to induce a significant IL-1β elevation in HD iMONOs. Presence of the heterozygous 1377delT *NFKB1* variant reduced levels of CXCL1 and CXCL2 in the CA01 background, with CXCL2 altered in both CA01 and HD backgrounds. All other cytokines measured were not significantly altered by introduction or correction of the 1377delT *NFKB1* variant into iMONOs from CA01 or HD, except CXCL5, which was decreased by the 1377delT variant in HD, but not CA01, iMONOs (Supplemental Figure 4).

Regarding broader genetic effects (i.e., those other than due to the *NFKB1* variant), we found that LPS-stimulated iMONO release of IL-12, as well as the chemokines CCL4 and CCL22, were elevated in cultures of CA01-derived iMONOs compared with those derived from HD (Figure 5B). However, levels of IL-12, CCL4, and CCL22 were unchanged after correction of the heterozygous 1377delT *NFKB1* variant in CA01 iMONOs or introduction of the variant into HD iMONOs. We confirmed this finding using a second HD iPSC line (data not shown). Elevation of CCL22 was also noted in CA01 relative to HD iMONO cultures with and without LPS stimulation but was not affected by correction or introduction of 1377delT *NFKB1*. CCL1 was elevated in unstimulated CA01 relative to HD iMONOs only, and CXCL5 was reduced in LPS-stimulated CA01 versus HD iMONOs and with introduction of 1377delT *NFKB1* into HD iMONOs only (Supplemental Figure 4). These results indicate that some aspects of CVID inflammatory dysregulation, including IL-12 elevation, may result from factors independent of the heterozygous *NFKB1* variant.

### Inflammatory pathway gene expression changes due to 1377delT NFKB1 variant in iMONOs derived from CA01 and HD.

**Figure 6:**
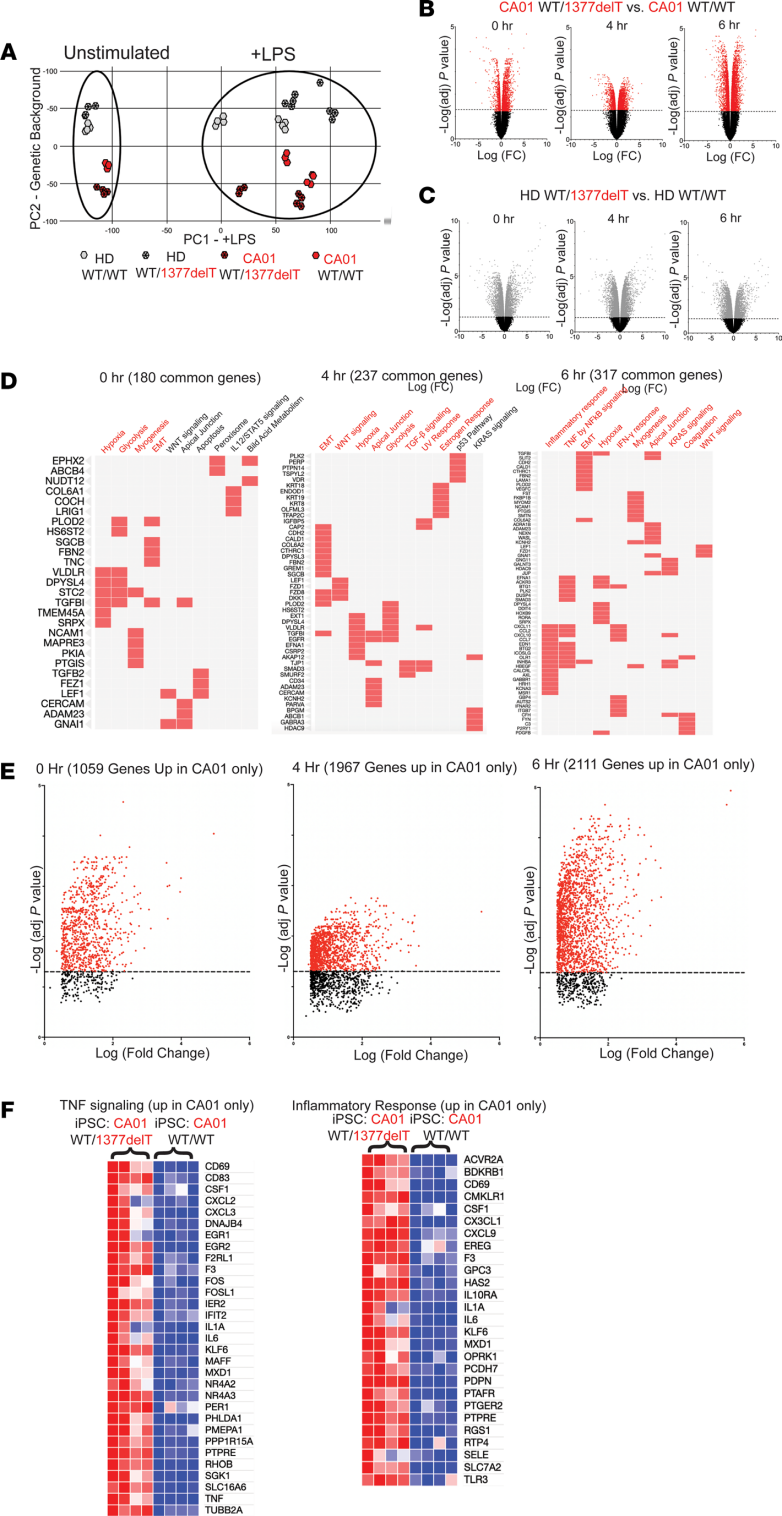
Inflammatory pathway gene expression changes due to 1377delT NFKB1 variant in iMONOs derived from CA01 and HD. (**A**) PCA plot of RNA-seq from iMONO cultures for CA01 and HD with and without heterozygous 1377delT NFKB1 variant. (**B** and **C**) Volcano plots illustrating distribution of RNA-seq changes from iMONO cultures with and without heterozygous 1377delT NFKB1 variant in CA01 (**B**) and HD backgrounds (**C**). (**D**) Top 10 affected pathways by *P* value from HALLMARK pathway clustergrams of gene upregulated by heterozygous 1377delT NFKB1 variant in iMONOs from CA01 and HD genetic backgrounds 0, 4, and 6 hours culture with LPS. EMT, epithelial mesenchymal transition. (**E**) Gene expression increased by 1377delT NFKB1 in CA01, but not HD, iMONOs. (**F**) Genes from TNF signaling and inflammatory response gene sets with increased expression due to heterozygous 1377delT NFKB1 in CA01, but not HD, iMONOs. Significant pathways in red. PCA, principal component analysis.

To understand the transcriptional dynamics preceding cytokine release and based on qPCR analysis of several marker genes of interest over time after culture with LPS, 4 and 6 hours were selected as time points for bulk RNA-seq (Supplemental Figure 5). All 4 iPSC lines were included in 0 hours, 4 hours, and 6 hours iMONO cultures with LPS, with 4 biological replicates, defined as separate differentiations of iMONOs. A principal component analysis (PCA) plot of our RNA samples demonstrated clusters along the 2 major sources of variance in our data set, culture condition (with or without LPS), and iPSC genetic background (CA01 or HD) (Figure 6A). Thus, like cytokine and chemokine protein levels measured in media of iMONO cultures, iMONO mRNA expression is influenced by iPSC genetic differences and inflammatory conditions, in addition to presence of the heterozygous *NFKB1* variant.

Focusing on gene expression changes due to the 1377delT *NFKB1* variant, we identified differentially expressed genes in iMONOs between isogenic lines at 0, 4, and 6 hours of culture with LPS using bulk RNA-seq. Isogenic comparison with *NFKB1* variant–corrected iMONOs in the CA01 background found 3,195 differentially expressed genes before culture, 3,629 genes after 4 hours of culture with LPS, and 5,806 genes after 6 hours of culture with LPS due to presence of the heterozygous 1377delT *NFKB1* variant (Figure 6B). Introduction of heterozygous 1377delT *NFKB1* in HD iMONOs resulted in significant differential expression of 5,966 genes before culture, 7,608 genes after 4 hours culture with LPS, and 5,327 genes after 6 hours of culture with LPS (Figure 6C). By isolating a list of genes with similar responses in both of these isogenic comparisons, we identified gene set pathways affected by the 1377delT *NFKB1* variant in both CA01 and HD background iMONOs using ENRICHR and analyzed against the HALLMARK pathway gene sets (Figure 6D). Without culture with LPS, gene sets upregulated by heterozygous 1377delT *NFKB1* across different genetic backgrounds include those relating to hypoxia, glycolysis, myogenesis, and epithelial to mesenchymal transition. After 4 hours of culture with LPS, additional pathways like WNT and TGF-β signaling are upregulated. Consistent with the observation that longer cultures of our iMONO system exposes greater differences in inflammatory response, 6 hours of culture with LPS revealed differences in gene sets representing inflammatory response, TNF signaling by NF-κB, and IFN-γ response (log_10_ *P* value of 1.33 × 10^–7^, 7.54 × 10^–7^, and 1.38 × 10^–3^, respectively).

Regarding gene expression changes due to the 1377delT *NFKB1* variant found in isogenic comparisons of CA01 but not HD iMONOs, we found 1,059 genes upregulated after 0 hours, 1,967 genes up after 4 hours, and 2,111 genes up after 6 hours (Figure 6E). Again, of the 3 time points we examined, 6 hours of culture with LPS caused upregulation of the most number of genes relating to inflammatory gene expression, including increases in gene sets representing inflammatory response and TNF signaling via NF-κB. Notably, genes upregulated by heterozygous 1377delT *NFKB1* variant in CA01, but not HD, iMONOs included the NF-κB–driven cytokines IL-1α, IL-6, and TNF as well as the neutrophil chemoattractants CXCL2 and CXCL3 (Figure 6F). These results indicate key inflammatory genes and pathways altered by the heterozygous 1377delT *NFKB1* variant more in the patient with CVID than HD.

### Clinical response to TNF antagonism in patient CA01 corresponds with resolution of ulcerative neutrophilic gastritis.

**Figure 7:**
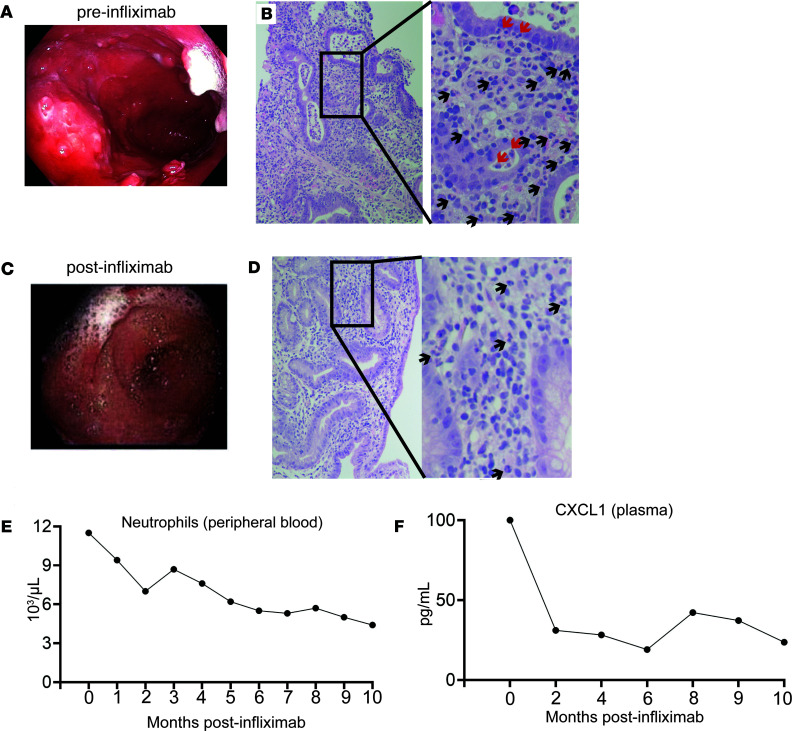
Clinical response to TNF antagonism in patient CA01 corresponds with resolution of ulcerative neutrophilic gastritis. (**A**) Endoscopic image of stomach with diffuse ulcerations. (**B**) Biopsy from the gastric body shows active chronic gastritis, atrophy, and absence of parietal cells (H&E stain at 200× and 400× [inset] magnification). Abundant neutrophils are seen in the lamina propria (black arrows) and within the epithelium, forming crypt abscesses and cryptitis (red arrows). (**C**) Endoscopic image of stomach showing resolution of diffuse ulcerations after infliximab. (**D**) Endoscopic biopsy of stomach body after infliximab shows fewer neutrophils in the lamina propria (black arrows), with resolution of cryptitis and crypt abscesses. (**E**) Effect of infliximab on CA01 peripheral neutrophil count over time. (**F**) Effect of infliximab on CA01 plasma CXCL1 over time.

Repeat endoscopic biopsy found no change in intraepithelial lymphocytosis of the small bowel despite marked clinical improvement after TNF antagonism in patient CA01. Notably, severe gastritis with mucosal ulcerations, absence of parietal cells, and neutrophil-predominant inflammation within the epithelium, forming crypt abscesses and cryptitis, was present prior to TNF antagonist therapy (Figure 7, A and B). Gastric biopsy had negative gastrin and *Helicobacter pylori* immunostain (data not shown). This ulcerative neutrophilic gastritis improved with infliximab treatment, including resolution of gastric ulcerations (Figure 7C), cryptitis, and crypt abscesses, as well as fewer neutrophils in the lamina propria (Figure 7D). Convalescence of neutrophilic gastritis corresponded with reduction of neutrophils in the peripheral blood (Figure 7E), while levels of circulating lymphocytes and monocytes were unchanged (data not shown). Plasma levels of the neutrophil chemoattractant chemokine CXCL1 was also reduced by infliximab (Figure 7F). However, there was no significant reduction in other plasma proteins measured, included the systemic inflammatory marker soluble CD14 (data not shown). These results suggest that efficacy of infliximab in patient CA01 may be related to the role of TNF in neutrophil recruitment and/or NF-κB–related inflammation potentiated by the heterozygous 1377delT *NFKB1* variant in the GI tract.

## Discussion

We applied an iPSC model, together with CRISPR-mediated gene editing, to study immunological effect of a heterozygous frameshift *NFKB1* variant found in a patient with CVID with a severe clinical course that was responsive to TNF antagonism. This approach allowed, for what we believe to be the first time, exploration of how a heterozygous *NFKB1* variant affects inflammatory cytokine production and gene regulation in a purified cell type in 2 distinct genetic backgrounds. While the heterozygous 1377delT *NFKB1* variant caused similar protein haploinsufficiency in both CVID- and HD-derived iMONOs, its effect upon inflammatory response was greater in iMONOs derived from the patient with CVID in which the variant was identified. Furthermore, despite being an NF-κB–driven cytokine, IL-12 elevation was observed in the CVID patient’s iMONOs, independent of the heterozygous *NFKB1* variant, as correction of the variant did not ameliorate its elevation. While previously recognized as frequent genetic etiology of CVID and one that predisposes to a more inflammatory disease course, our results paint a more complex picture of heterozygous *NFKB1* variants than previously understood, one shaped by *NFKB1*-independent factors and variation between individuals.

Importantly, we were able to produce iMONOs that were morphologically uniform, equally viable, and with similar surface markers from CVID patient iPSCs and HD-derived iPSCs with and without the heterozygous 1377delT *NFKB1* variant. Moreover, the heterozygous 1377delT *NFKB1* variant similarly reduced proteins levels of both p105 and p50 in iMONOs derived from CVID or HD iPSCs, under unstimulated and LPS-stimulated conditions. As the other NF-κB family members were not significantly affected, this represents strong evidence that heterozygous *NKFB1* 1377delT results in haploinsufficiency of p105/p50. This is consistent with previous reports implicating haploinsufficiency as the major consequence of human *NFKB1* variants (16). One possible driver of inflammatory disease in this patient could be inadequate levels of regulatory p50 homodimers, due to the observed p50 haploinsufficiency. Transcripts from the 1377delT *NKFB1* allele may be degraded before they are translated, or a defective protein product may be rapidly eliminated, thus producing haploinsufficiency of both p105 and p50. Analysis of *NFKB1* transcripts by qPCR did not reveal a significant reduction as could be indicative of nonsense mediated decay, but such analysis does not definitively rule out this potential driver of haploinsufficiency (34). Ability to investigate the involvement of noncoding RNAs in this regulation was limited by our use of PolyA selection. No truncated product of 1377delT *NFKB1* was detected. An advantage of the endogenous gene expression of iPSC-based model we utilized over studies with cellular overexpression systems using cDNA may be the presence of introns that mediate posttranscriptional regulation, such as nonsense-mediated decay. As a result, the iPSC model is less likely to result in truncated proteins that may be artifacts of a cDNA overexpression system. Further efforts are needed to more firmly demonstrate how the 1377delT *NFKB1* variant causes haploinsufficiency and promotes inflammatory disease.

Using the iMONO system, we found that LPS stimulated supernatant levels of IL-1β were significantly elevated by heterozygous 1377delT *NFKB1* variant in iPSCs generated from the patient with CVID but not that from a HD. This result indicates that the effect of *NFKB1* variants upon cytokine production may vary between individuals. Protein levels of CXCL1 and CXCL2 were significantly reduced by the 1377delT *NFKB1* variant in CVID-derived iMONOs, with CXCL2 also reduced when the variant was present in HD-derived iMONOs. This was a puzzling result as supernatant protein and RNA from culture had conflicting results. While mechanism for this discrepancy is unclear, microRNAs have been shown to reduce CXCL1 protein expression without affecting levels of its mRNA (35).

IL-12, CCL4, and CCL22 were all elevated in iMONOs derived from CA01, but unrelated to presence of the 1377delT *NFKB1* variant, indicating that other aspects of a patient with CVID may cause cytokine dysregulation. It was surprising that protein measurement for relatively few NF-κB–driven cytokines were altered by the heterozygous frameshift *NFKB1* variant we studied, particularly given the greater differences we detected via RNA-seq. In its current form, our iMONO system may not fully model cytokine protein dysregulation imparted by an *NFKB1* variant at the protein level. Relatively high levels of LPS (100 μg/mL) and duration of culture (16 hours) were required to expose an effect of the heterozygous *NFKB1* variant upon IL-1β and IL-12 in our system. Trained immunity or additional priming, such as with IFN-γ, may enhance cytokine protein release, more closely model monocytes from the inflammatory milieu of CVID, and better align protein results with that of RNA-seq (36, 37). Further exploration of the iMONO model, and how it may best be used to study CVID-related inflammation, is needed.

Cytokines we found elevated in the iMONO system have noted significance to CVID. We found heterozygous 1377delT *NFKB1* necessary for elevation of IL-1β release by LPS stimulated CA01-derived iMONOs. IL-1β is a principal cytokine released upon inflammasome activation, is a cornerstone of the inflammatory response, and, of particular relevance given the clinical course of the patient, can be a key component of GI inflammation (38–40). Moreover, loss of NFKB1 has been associated with gastric inflammation and cancer in mice (41, 42). Human frameshift *NFKB1* variants have been reported to promote inflammasome activation, though subjects in that study did not meet CVID diagnostic criteria (14). The role of inflammasome activation and IL-1β in CVID, including those with *NFKB1* variants, remains a significant knowledge gap that our iPSC-based platform could address in future studies. Notably, we also found IL-12 release to be elevated in the iMONO system, but this elevation was independent of the heterozygous *NFKB1* variant. IL-12 has been previously found elevated in CVID with inflammatory complications, including its production from monocytes, and is a central driver of type 1 (IFN-γ) inflammation that characterize these patients (24, 27, 43, 44). Our results further justify ongoing efforts to understand the significance of increased type 1 immune responses in CVID.

To gain broader insight into the effect of the heterozygous 1377delT *NFKB1* variant upon inflammation, we examined its effect upon transcription in the iMONO system. While PCA unsurprisingly found presence or absence of LPS to be a primary component of the RNA-seq results, the secondary component was genetic background of the iPSC line (CVID or HD) rather than presence or absence of the *NFKB1* variant. This was supportive of our results at the cytokine level and highlighted the importance of *NFKB1*-independent factors in shaping the effect of a heterozygous *NFKB1* variant. These results led us to more closely examine the direct effect of the heterozygous 1377delT *NFKB1* variant by defining the gene expression changes that were shared, as well as differed, between both genetic backgrounds when the variant was present. After 6 hours of culture with LPS, presence of heterozygous 1377delT *NFKB1* variant significantly altered gene expression of pathways consistent with the inflammatory phenotype of the patient, including numerous components of the inflammatory response, TNF signaling by NF-κB, and IFN-γ response gene sets. In CVID patient–derived iMONOs, the 1377delT *NFKB1* variant had a greater effect on mRNA expression of genes encoding the NF-κB–driven cytokines IL-1β, IL-6, and TNF as well as the neutrophil chemoattractants CXCL2 and CXCL3. Such results further our understanding of how *NFKB1*-independent factors that vary among individuals may shape the effect of heterozygous *NFKB1* variants, potentiating inflammatory dysregulation in a manner that may be relevant when selecting a therapeutic agent.

While our study did not uncover a simple explanation for the potency of TNF antagonism in this patient, we did find evidence that, at the RNA level, the heterozygous 1377delT *NFKB1* variant increased expression of *TNF*, TNF-related NF-κB signaling, and the inflammatory response upon stimulation with LPS, which signals via NF-κB. Additionally, we found that heterozygous 1377delT *NFKB1* elevated IL-1β release from iMONOs derived from the patient with CVID. TNF antagonism can reduce priming of the inflammasome, potentially reducing inflammasome-derived cytokines like IL-1β, but it is unclear if this was the primary mechanism for the clinical improvement of our patient (45–47). Notably, the 1377delT *NFKB1* patient with CVID did have resolution of ulcerative neutrophilic gastritis after TNF blockade. TNF is a potent inducer of neutrophil chemoattractant chemokines, and we did see reduction of CXCL1 in the plasma over time upon initiating treatment with infliximab as well as increased mRNA expression of the neutrophil chemoattractants CXCL2 and CXCL3 due to the 1377delT heterozygous *NFKB1* variant in CA01-derived iMONOs (48–50). Thus, the role of TNF antagonism in reducing neutrophilic inflammation may be an important factor contributing to the patient’s response to infliximab. It must also be noted, however, that since gastritis may occur in CVID in the absence of a heterozygous *NFKB1* variant, as can clinical response to TNF antagonism, it is possible that the GI disease occurring in this patient is the result of *NFKB1*-independent factors.

While providing insight regarding effect of a heterozygous *NFKB1* variant, and how *NFKB1*-independent factors shape this effect, there are limitations to our approach. An extensive initial resource investment of cost, effort, and time was needed to establish this model, limiting us to study of a single variant. While our results may be indicative of what to expect from other nonsense or frameshift *NFKB1* variant that cause haploinsufficiency, we cannot be certain. However, the efficiency of CRISPR-Cas9 gene editing may allow our group, as well as others, to expand iPSC-mediated studies to involve greater numbers CVID *NFKB1* variants and further close this knowledge gap. Such future studies could involve differentiation of iPSCs into other cell types, to expand our understanding of *NFKB1* variant effect. Furthermore, coculture or organoid studies would help provide a more complex understanding of these variants with potentially fewer limitations than an iMONO single-cell type system. This could include culture of iMONOs with other immune cells or generation of gastric organoids to model the gastritis observed in our patient.

Correction of the heterozygous 1377delT *NFKB1* variant did not resolve certain cytokine elevations, and introduction of the heterozygous *NFKB1* variant did not reproduce all the findings of patient-derived iMONOs. Thus, the heterozygous *NFKB1* variant may act within a digenic or more blended genetic contribution to disease. While whole exome sequencing did not reveal other candidate pathogenic variants, whole genome analysis may have revealed additional potential genetic contributors. Unfortunately, we were unable to perform this additional level of genomics analysis. Digenic and polygenetic etiologies of CVID have been suggested, including heterozygous *TNFRSF13B* variants, which are frequently found in CVID but accepted to be disease modifiers rather than monogenic etiologies (51–55). Perhaps in our patient, the heterozygous *NFKB1* 1377delT variant is acting with other genetic factors or etiologies to promote the CVID phenotype observed. Indeed, we have found that heterozygous *NFKB1* variants, frameshift and nonsense in particular, promote inflammatory disease in CVID (15).

Heterozygous *NFKB1* variants are thought to be one of the most frequent genetic etiologies of CVID. Applying an iPSC- and CRISPR/Cas9-mediated model system to CVID, for what we believe to be the first time, our data paint a complex picture of *NFKB1* variants in CVID, one shaped by *NFKB1* variant–independent factors, which can lead to pathogenic inflammation, such as neutrophilic gastritis in our study patient. While the results highlight strengths of an iPSC-based approach to study genetic variants in inborn errors of immunity, future studies leveraging more complex cellular interactions, such as inflammatory priming, coculture with other cells, or production of organoid models, may further our understanding of how *NFKB1* variants affect CVID. Continuing efforts to model immune dysregulation and disease manifestations aligned with data from the medical record may improve how iPSCs are deployed to study complex immune disorders like CVID.

## Methods

### Sex as biological variable.

Both female and male patients were recruited for this study. For the purposes of this study, no differences between female and male patients were noted.

### Patient records.

Patient age, sex, laboratory values, and clinical history were obtained from electronic medical records.

### Isolation of PBMCs and plasma.

PBMCs and plasma were isolated using Ficoll-Paque density gradient centrifugation at 400 × *g*. Plasma was stored at –80°C, and PBMCs were stored at –135°C.

### iPSC generation and CRISPR-Cas9 editing.

iPSCs were generated from PBMCs using the CytoTune-iPS 2.0 Sendai Reprogramming kit (ThermoFisher) at the Center for Regenerative Medicine of Boston University and Boston Medical Center iPSC Core Facility (19). iPSCs were validated by via karyotyping, immunofluorescence staining of the human pluripotent stem cell markers TRA-1-60 (Sigma, MAB4360) and TRA-1-81 (Sigma, MAB4381), verification of ability to develop into each of the 3 germ layers (ectoderm, endoderm, mesoderm), and presence of heterozygous *NFKB1* variant by sequencing. The CRISPR (clustered regularly interspaced short palindromic repeats)/Cas (CRISR-associated) system was used for gene editing iPSCs (20–22, 56). Bacteria containing the Cas9 plasmid (Addgene) was grown in selection media and plasmid DNA extracted using Maxiprep (Qiagen). Sequence analysis software was used to identify the genomic target sequence (gRNA) with minimal alternate targets and correct the *NFKB1* variant to WT. This gRNA fragment (5′–3′) (UUUCAAUAACUUUCCCAAAGAGG for variant introduction, GUUUCAAUAACUUUCCCAAGAGG for variant correction) was synthesized commercially and cloned into a pCRII-TOPO vector (ThermoFisher), expanded in chemically competent *E*. *coli*, and then subjected to confirmatory Sanger and next generation sequencing. Next gen sequencing was used to determine whether the variant and WT transcripts are present in equal ratios expected from heterozygosity. iPSCs cultured on Matrigel-coated plates were transfected with this correction vector via electroporation. Correction of *NFKB1* variant and absence of off-target effects of gene-editing approach was confirmed by Sanger sequencing. CHOPCHOP software was used to design the most effective guides and to predict potential off target edits. Sanger sequencing of PCR-amplified fragments was then completed after editing, to ensure no off-target edits were present in the top 20 predicted off-target sites for the selected guide. iPSCs were harvested using Gentle Cell Dissociation Reagent (StemCellTech). Custom crRNAs were hybridized to Alt-R CRISPR-Cas9 tracrRNAs at 95°C for 5 minutes. Hybridized gRNA complex was then incubated with Cas9 Nuclease V3 (IDT) at room temperature for 10–20 minutes. Harvested iPSCs were put into single cell suspension in CloneR 2 (StemCellTech) RNP complexes, Alt-R Cas9 Electroporation enhancer (IDT), and custom ssODN HDR Donor templates (IDT) were added to 1 × 10^6^ cells in suspension and nucleofected using a Lonza 4D-Nucleofector Unit (program CB150). Nucleofected cells were immediately resuspended in Cloner2 supplemented mTESR and plated on prewarmed Matrigel (Corning) coated plates with HDR enhancer (IDT) at a concentration of 25,000 cells per 6 wells. Cells were cultured overnight at 37°C, 5% CO_2_. Media was changed the next day to remove HDR enhancer. Media were not changed for a week as colonies were allowed to emerge. Once they reached sufficient size, individual colonies were then manually scraped into individual 96-well Matrigel-coated plates. Clones were expanded in mTESR and passaged onto replicate plates. One replicate plate was used for mutation screening by applying gDNA QuickExtract (Thermo) and using the sanger sequencing protocol described above. Clones positive for editing were expanded and karyotyped (CellLineGenetics). Indel analysis was performed by Sanger sequencing of 100 base pairs around the top 10 off target sites predicted by CHOPCHOP to ensure no off target edits at these sites.

### Sanger sequencing.

gDNA samples were extracted from cells using DNeasy Blood and Tissue Kit (Qiagen). Two hundred base pairs of NFKB1 Exon 14 were amplified using custom primers (IDT) and Q5 high fidelity PCR master mix (NEB). Amplicons were sequenced using sanger sequencing from GeneWiz.

### iPSC culture.

iPSCs were cultured under feeder free conditions using Matrigel (Corning) coated tissue culture plates (Corning) and mTESR (StemCellTech) supplemented with Primocin (Invivogen). Media was changed daily. Cells were cultured to 60%–80% confluency prior to passage. Cells passaged with RelesR (StemCellTech) were plated at a 1:100 dilution in fresh wells with mTESR supplemented with ROCK inhibitor (StemCellTech).

### iMONO differentiation and culture.

On Day 0, iPSCs were harvested using Versene (Gibco) and plated on ultralow attachment U bottom 96-well plate (Corning) at a concentration of 10,000 cells per well. Each well contained 50 μL of mTESR supplemented with 50 ng/mL BMP4 (R&D), 20 ng/mL SCF (R&D), 50 ng/mL VEGF (R&D), and 10 μM ROCK inhibitor (StemCellTech). On Day 2, an additional 50 μL of Day 0 media without ROCK inhibitor was added to each well. On Day 4, EB were mechanically harvested and transferred to a gelatin (Gibco) coated flask at a concentration of 96 EBs per T75 in Xvivo15 Media (Lonza), supplemented with 2 mM Glutamax (Gibco), 0.55 mM B-mercapthoethanol (Gibco), 100 ng/mL mCSF (R&D), and 25 ng/mL IL-3 (R&D). Flasks were cultured undisturbed at 37°C, 5% CO_2_ for a week. Media were changed weekly until the appearance of iMONOs around week 3.

### iMONO flow cytometry validation.

iMONOs were centrifuged at 400 × *g* and resuspended in 1 mL FACS buffer (DPBS, 10% FBS, 2 mM EDTA, Gibco). Live/Dead stain (Invitrogen LIVE/DEAD) was added to fully stained samples and allowed to sit on ice for 20 minutes in the dark. Cells were then washed with FACS buffer and blocked with FC Block (Thermo). Antibodies were suspended in Horizon Brilliant Buffer Solution Plus (Thermo), and antibody master mix was added to cells for 60 minutes on ice in the dark. Cells were washed 3 times with FACS buffer and strained directly into FACS tubes. Samples were run on a 5-laser Cytek Aurora. FITC CD14 (BioLegend, HCD14), APC-Cy7 CD45 (BioLegend, 2D1), PE-Fire 640 HLA-DR (BioLegend, I243), Alexa Flour 647 CX3CR1 (BioLegend, 2A9-1), PE CD163 (BioLegend, GHI/61), PerCP-Vio 700 CD16 (Miltenyi Biotec, REA423), PE-Cy7 CD11b (Invitrogen, ICRF44), BUV 737 CCR2 (BD Biosciences, LS132.1Dg), and BV421 CD64 (BD Biosciences, 10.1).

### Protein/RNA/supernatant extraction.

iMONOs were harvested from their differentiation flask and passed through a 40 μm filter. Cells were spun down and plated on tissue culture treated wells at a concentration of 400,000 cells per mL in Immunocult-SF Macrophage Media (Stem Cell Tech) supplemented with 100 ng/mL mCSF (R&D). Cells were cultured overnight at 37°C, 5% CO_2_. Ultrapure LPS (Invitrogen) was then added to cultures as needed. At the end of treatment, supernatants were harvested, spun at 20,000*g*, transferred to a new tube, and stored at –80*°*C. For protein extraction, cells were then mechanically scraped into ice-cold DPBS, washed, spun down, and lysed in RIPA buffer (Thermo) supplemented with protease inhibitors (Sigma) for 20 minutes on ice. Extracts were then spun at 20,000*g*, transferred to a new tube, and stored at –80°C. For RNA extraction, RNeasy Micro Kit was used (Qiagen) and samples were stored at –80°C.

### Western blot and densitometry.

Protein extract concentration determined by BCA assay. In total, 20 μg of protein were denatured at 70°C using NuPAGE LDS Sample Buffer (Thermo) and NuPAGE reducing agent (Thermo). Samples were then loaded onto a 3%–8% Tris Acetate gel with a Dual Chameleon Protein Ladder (Thermo) and run at 200V. Samples were then transferred to a nitrocellulose membrane (Thermo), which was imaged for total protein using a Total Revert Staining Kit (Licor). Membranes were then blocked with Blotto TBS (Thermo) and incubated with primary antibody at the recommended dilutions overnight at 4°C. The primary antibodies used were p105/p50 (Cell Signaling Tech, 3035S), RelB (Cell Signaling Tech, 4954S), C-Rel (Cell Signaling Tech, 4727S), p100/p52 (Cell Signaling Tech, 4882S), and p65 (Cell Signaling Tech, 3035S). The next day, membranes were washed in TBS, incubated in secondary antibody at the suggested dilutions for 1 hour at room temperature in the dark, and then washed again with TBS and imaged using a Licor Odyssey CLx Imager. Quantification performed using ImageStudio software. Both total protein stain and corresponding NF-κB family member stain raw Western blot image files were imported to ImageStudio 6.0. For each image, the individual channel for each stain was then selected, red for total protein and green for NF-κB specific stain, and the analysis function was used to position shapes measuring the enclosed signal within each lane of the image. For NF-κB family member specific stains, contrast and brightness settings were adjusted as needed to identify the appropriate positions of bands and analysis shape position but were then reverted back to their original raw settings for quantification. For each total protein stain, the lane normalization factor was then calculated by dividing the signal within each lane by the highest lane signal for the given image. Using the NF-κB family member specific stain, each band signal was then divided by its corresponding lane normalization factor from the total protein stain to produce a normalized signal for the band. Normalized band signals were then statistically compared using 1-way ANOVA.

### Protein cytokine and chemokine measurement.

Frozen plasma samples from CVID and HCs were thawed on ice and diluted 1:2 or 1:10 in PBS. Levels of IL-6 (R&D Systems, DY206), IL-12p40 (R&D Systems, DY1240), and TNF (R&D Systems, DY210) in plasma as well as TNF in PBMC culture supernatants were measured by ELISA. Levels of CCL1, CCL4, CCL5, CCL22, CXCL1, CXCL2, CXCL5, IL-1α, IL-1β, IL-12, IL-18, and TNF in iMONO supernatants were measured using a Luminex assay kit (R&D Systems) according to the manufacturer’s instructions. Ninety-six–well plates of 50 μL diluted plasma samples were analyzed on a Luminex Magpix system. A Luminex inflammatory cytokine and chemokine panel was selected that contained proteins, including IL-18 and TNF, previously reported to be elevated in plasma of patients with CVID and to be representative of key cytokine and chemokine responses from myeloid innate immune cells that might be altered by NF-κB dysregulation. Blood was drawn at regularly scheduled outpatient visits. iMONO supernatants were analyzed in a similar way using the same Luminex cytokine panel.

### qPCR/RNA-seq.

RNA samples were extracted using RNeasy Micro Kits (Qiagen). Probes for *NFKB1*, *NLRP3*, *IL1B*, *TNFA*, and *IL12* purchased from IDT and amplified using TaqMan RNA-to-Ct 1-step kit (Thermo). Library prep and bulk RNA sequencing was performed by the BU Microarray and Sequencing Core.

### RNA-seq data analysis.

RNA libraries were prepared using the NEB Ultra Express Kit with Poly-A selection. The quality of RNA samples submitted was high, as evidenced by a very low level of degredation, as all samples used for RNA-seq had RNA integrity numbers of 9 or 10. The quality of the raw data was assessed using FastQC v.0.11.7 (57). Paired-end RNA-seq was performed using the Illumina NextSeq2000. The sequence reads were aligned to the GRCh38 reference using STAR v.2.6.0 (58). Counts per gene were summarized using the featureCounts function from the subread package v.2.0.3 (59). The edgeR package v.4.2.0 (60) was used to import, organize, filter and normalize the counts and the matrix of counts per gene per sample was then analyzed using the limma/voom normalization method (61). Genes were filtered based on the standard edgeR filtration method using the default parameters for the “filterByExpr” function. After exploratory data analysis with PCA, contrasts for differential expression testing were done for each of the infected samples versus mock (controls) at each time point (days after infection) for the infected samples. Differential expression testing was also conducted to compare the gene expression between the 2 infected time points and to investigate the time-specific effects in response to infection. The limma package v.3.60.0 (61) with its voom method — namely, linear modeling and empirical Bayes moderation was used to test differential expression (moderate 2-tailed *t* test). *P* values were adjusted for multiple testing using Benjamini-Hochberg correction (FDR-adjusted *P* value). Differentially expressed genes for each comparison were visualized using Glimma v.2.14.0 (62), and FDR < 0.05 was set as the threshold for determining significant differential gene expression.

### Statistics.

For plasma measurement comparisons, the *P* value was calculated by 1-way ANOVA with Kruskal-Wallis test for multiple comparisons. For Western blot densitometry comparison, the *P* value was calculated by 1-way ANOVA with Holm-Šídák test for multiple comparisons. For PBMC and iMONO cell culture data, the *P* value was calculated by 2-way ANOVA with Holm-Šídák test for multiple comparisons. Statistical tests were considered significant at a *P* value less than 0.05.

### Study approval.

This study was approved by the IRB of the Boston University Chobanian & Avedisian School of Medicine, Boston, Massachussetts, USA. Participants provided written informed consent prior to participation in the study. Research abided by the Code of Ethics of the World Medical Association (Declaration of Helsinki).

### Data availability.

RNA-seq data are available from the NIH Gene Expression Omnibus (GEO) under the accession no. GSE311471. All other raw data files are available from the Supporting Data Values file or from the corresponding author upon request.

## Author contributions

KMH designed research studies, conducted experiments, acquired and analyzed data. KB, MLA, GG, and MSW conducted experiments and acquired and analyzed data. PJS provided methodology for monocyte differentiation from iPSCs. PB performed RNA-seq analysis. MJ derived iPSCs from peripheral blood and facilitated storage of cells at the iPSC core. HMK provided clinical review of pathology samples and analysis of pathology findings. CCR contributed to clinical care and data collection of patient CA01. JPM, GM, and DNK provided feedback on experimental design and data analysis. PJM conceived and secured funding for the project, designed research studies, and analyzed data. KMH and PJM wrote the manuscript. All authors reviewed and edited the manuscript.

## Funding support

This work is the result of NIH funding, in whole or in part, and is subject to the NIH Public Access Policy. Through acceptance of this federal funding, the NIH has been given a right to make the work publicly available in PubMed Central.

- NIH K23 AI137187 and NIH R21 AI151486
- Candace and Barry Sloane Charitable Foundation to PJM
- American Academy of Allergy Asthma & Immunology (AAAAI; to PJM)
- NIH T32 AI007035 (KMH)
- NIH R01HL095993, P01HL170952, and N01 75N92025R00004 (DNK and PJS)

## Supplementary Material

Supplemental data

Unedited blot and gel images

Supporting data values

## Version 1 03/23/2026

Electronic publication
