# Supplementary material for: Heterozygous NFKB1 variant causes inflammatory dysregulation shaped by broader genetic context in common variable immunodeficiency
Source: JCI Insight. 2026 Mar 23;11(6):e198703. doi: 10.1172/jci.insight.198703 (PMC13043090; doi:10.1172/jci.insight.198703)
Supplement: Supplemental data [file jciinsight-11-198703-s047.pdf]

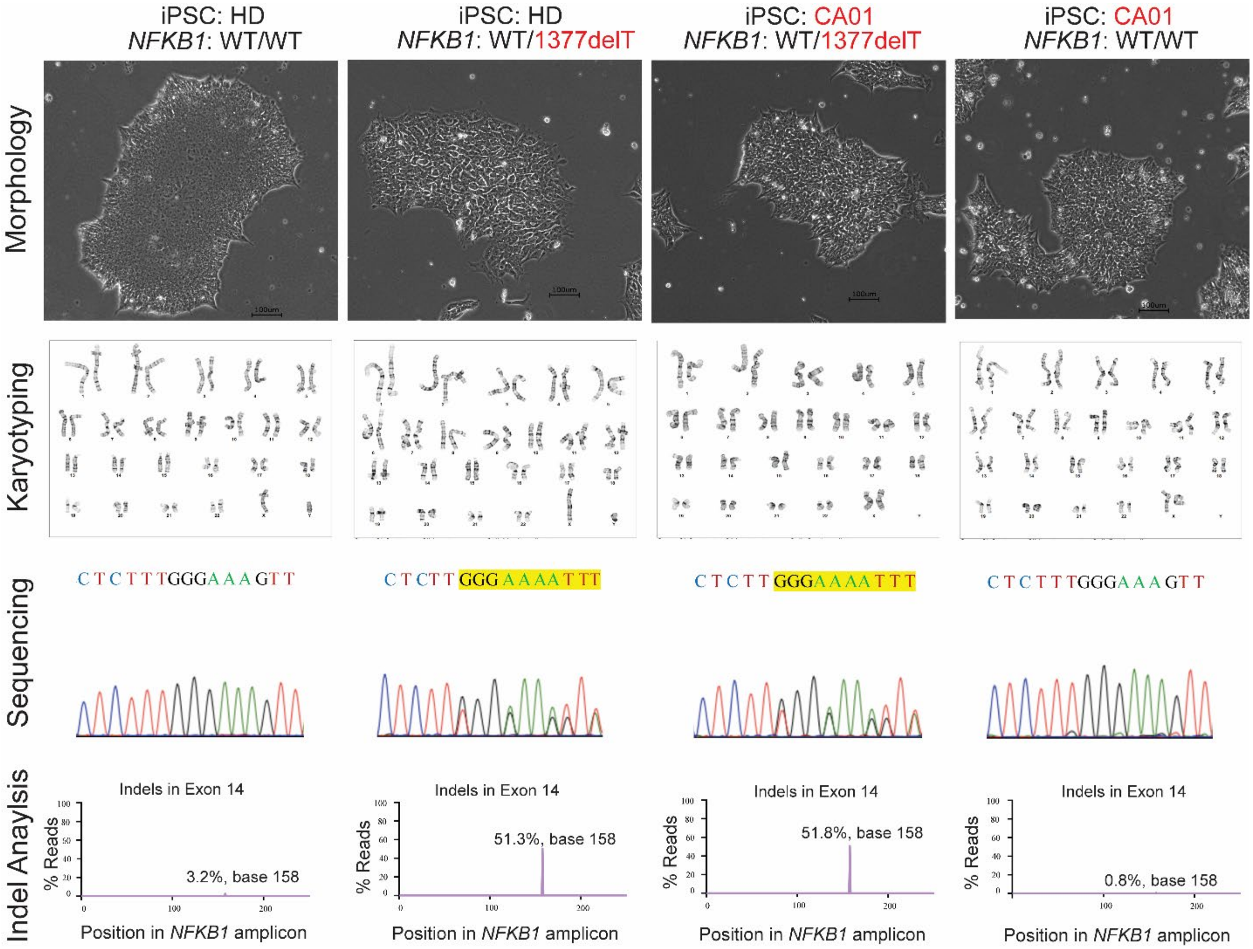

Supplementary Figure 1. iPSC lines used for this study show similar cell morphology, normal karyotyping, and expected *NFKB1* sequence. For each iPSC line: 10X brightfield image of iPSC in culture, G-banded karyotyping, Sanger sequencing chromatograms of gDNA amplified for *NFKB1* exon 14, and indel analysis of next generation sequencing from *NFKB1* exon 14 amplicon. Yellow highlight denotes heterozygous frameshift.

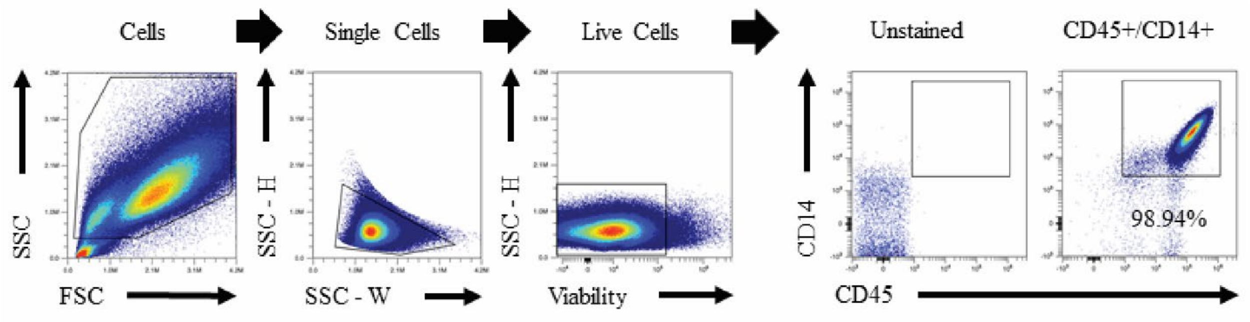

Supplementary Figure 2. Gating strategy for iMONO flow cytometry. Cells were gating on the basis of forward (FSC) and side (SSC) scatter, removal of putative cellular doublets, and collection of live cells using a viability dye. CD45+CD14+ cells were used for analysis of surface expression of markers expressed by monocytes (CD16 and CD163).

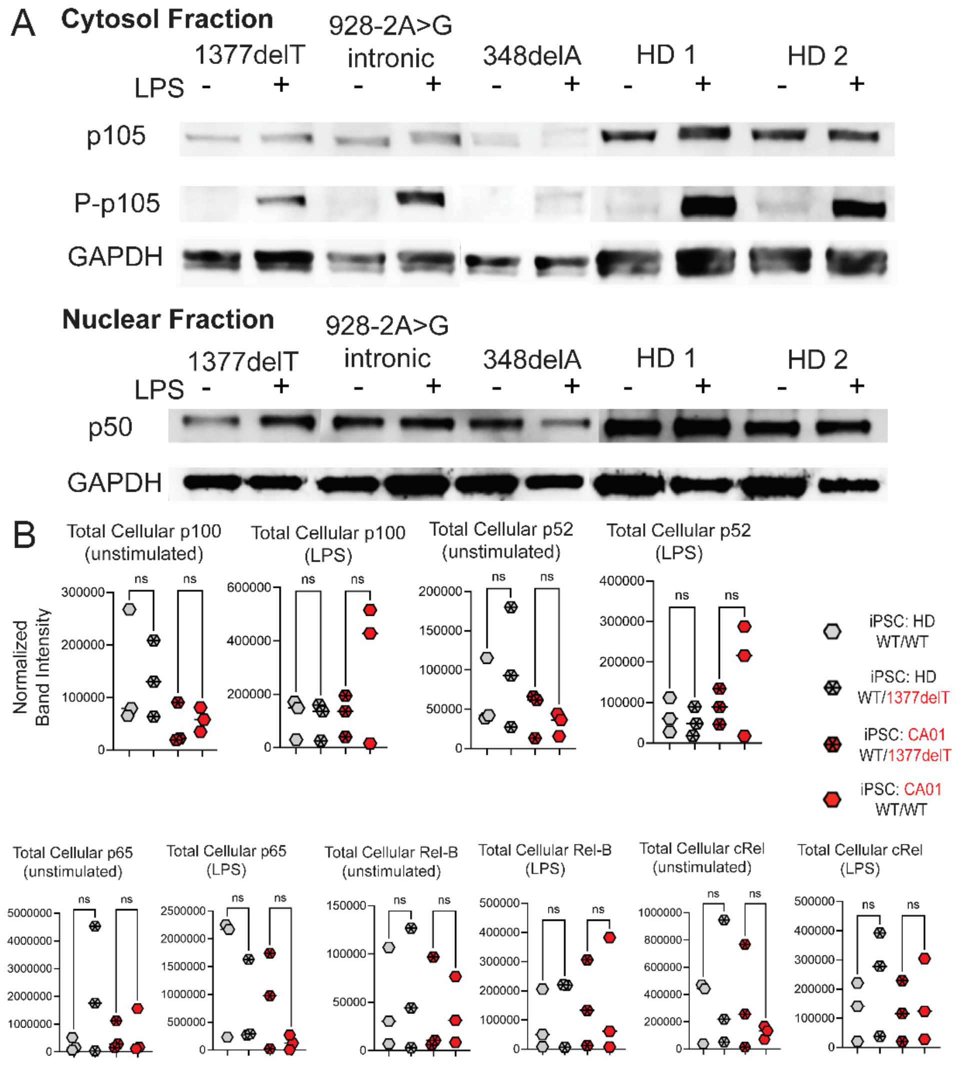

Supplementary Figure 3. *A*. Western blot of PBMCs from *NFKB1* variant subjects as indicated and two healthy donors (Control 1 & 2) after 18 hr culture with or without 20 ng/mL LPS. HD = healthy donor, GAPDH = protein loading control. *B*. Whole cell iMONO protein lysate (20 µg) analyzed for denoted protein by Western blot. Source of iPSC and presence of heterozygous *NFKB1* variant as indicated. Densitometry analysis of Western blot bands from three blots generated from three independent experiments. *P* value was calculated by one-way ANOVA with Holm-Sidak test for multiple comparisons.

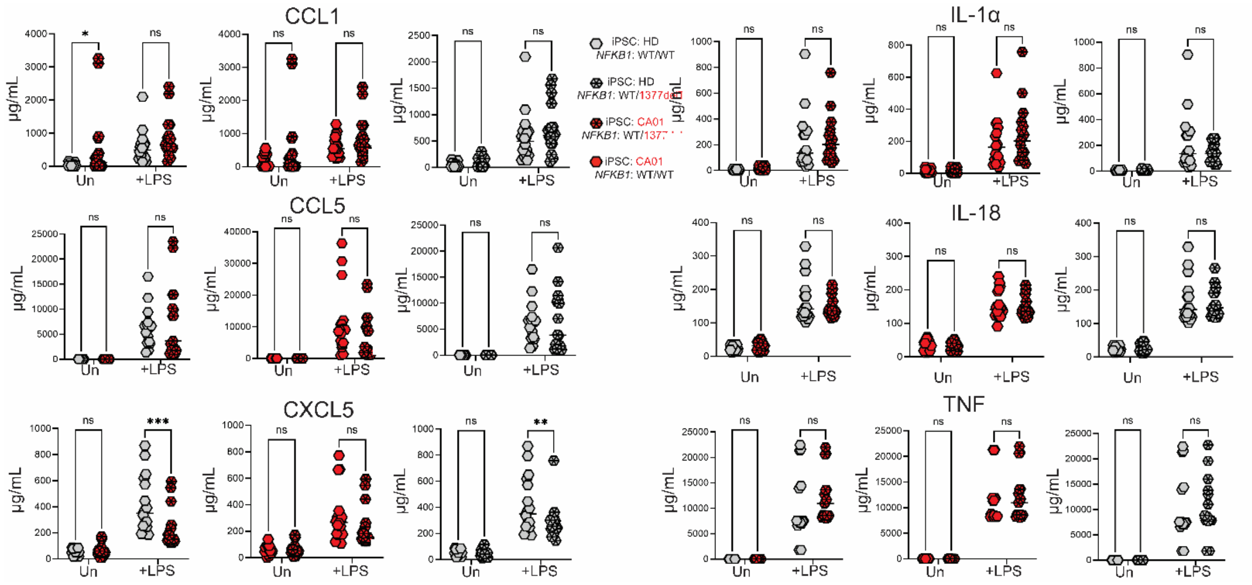

Supplementary Figure 4. Cytokine levels in iMONO culture supernatants with and without 18 hr culture with LPS. Parental iPSC line (CA01 or HD) and presence of WT or 1377delT *NFKB1* for both alleles as noted. *P* value was calculated by two-way ANOVA with Holm-Sidak test for multiple comparisons. * $P < 0.05$, ** $P < 0.01$, *** $P < 0.001$.

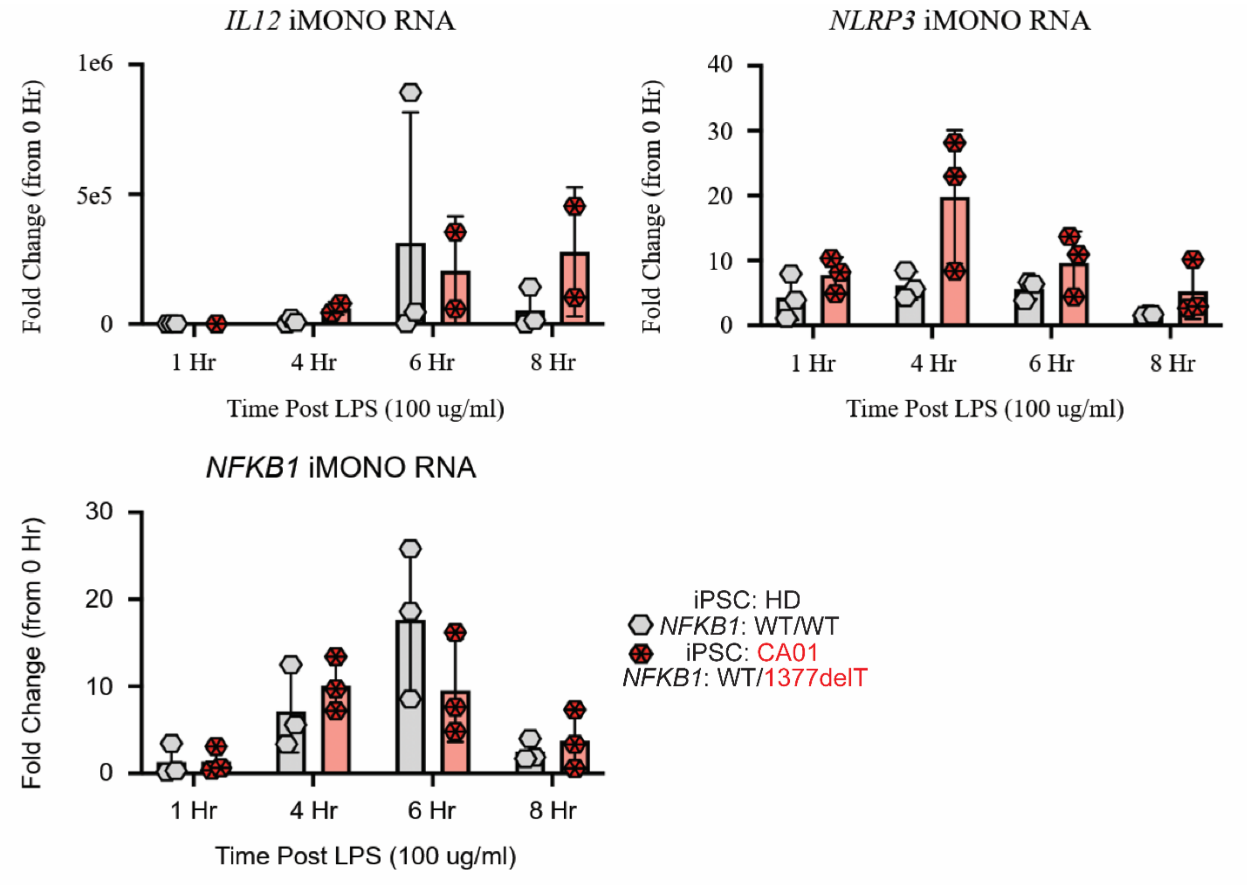

Supplementary Figure 5. Fold change measured by RT-qPCR and normalized to *GAPDH* expression by iMONOs of designated genes. Times listed denote culture with LPS. Data from CA01 iMONOs with 1377delT *NFKB1* variant shown as red, data from HD iMONOs without *NFKB1* variant shown as gray.
