# Supplementary material for: Heterozygous NFKB1 variant causes inflammatory dysregulation shaped by broader genetic context in common variable immunodeficiency
Source: JCI Insight. 2026 Mar 23;11(6):e198703. doi: 10.1172/jci.insight.198703 (PMC13043090; doi:10.1172/jci.insight.198703)

Data for Figure 4C. p50/p105Replicate Blot 1 (Including densitometry boxes and signal values)

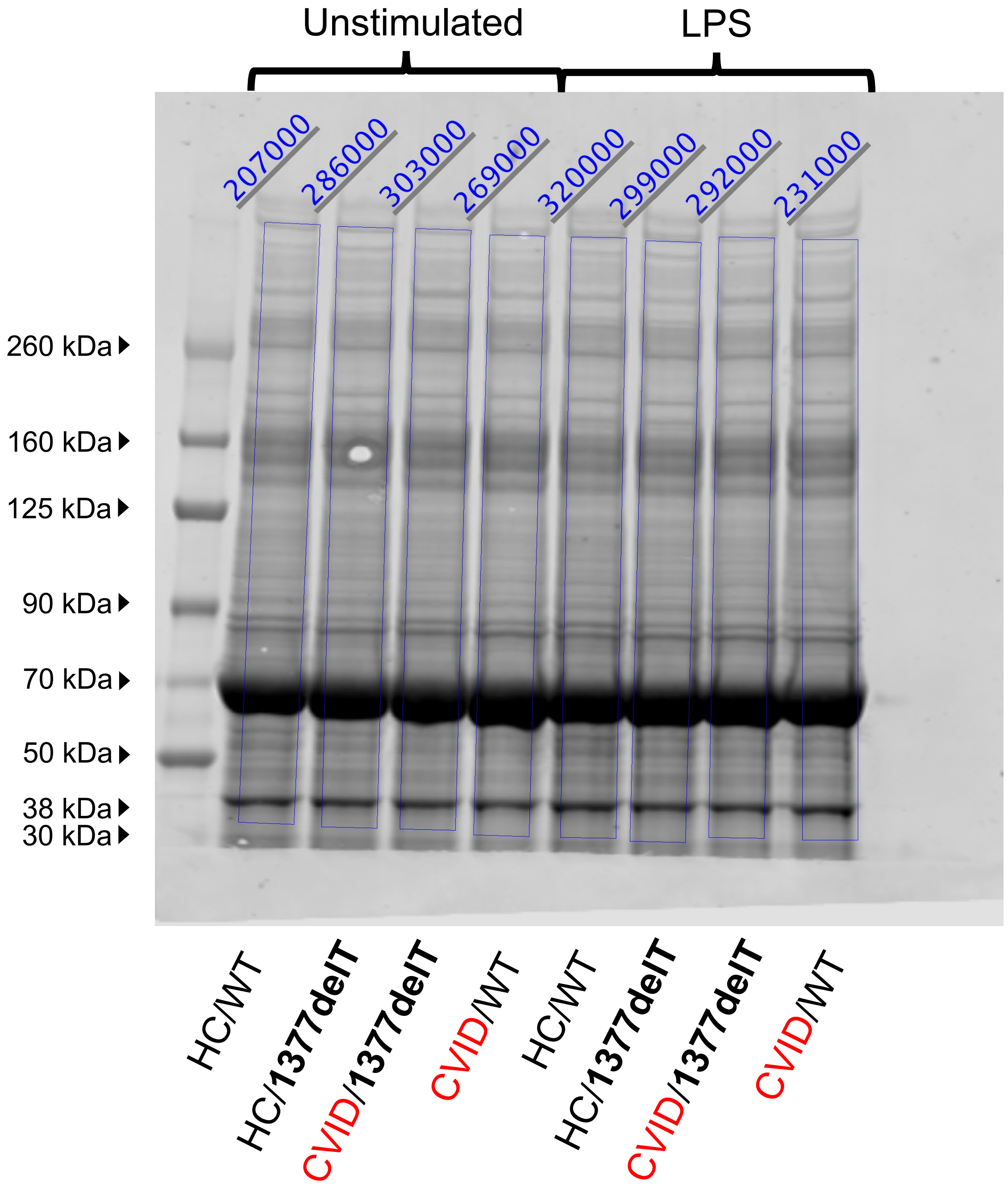

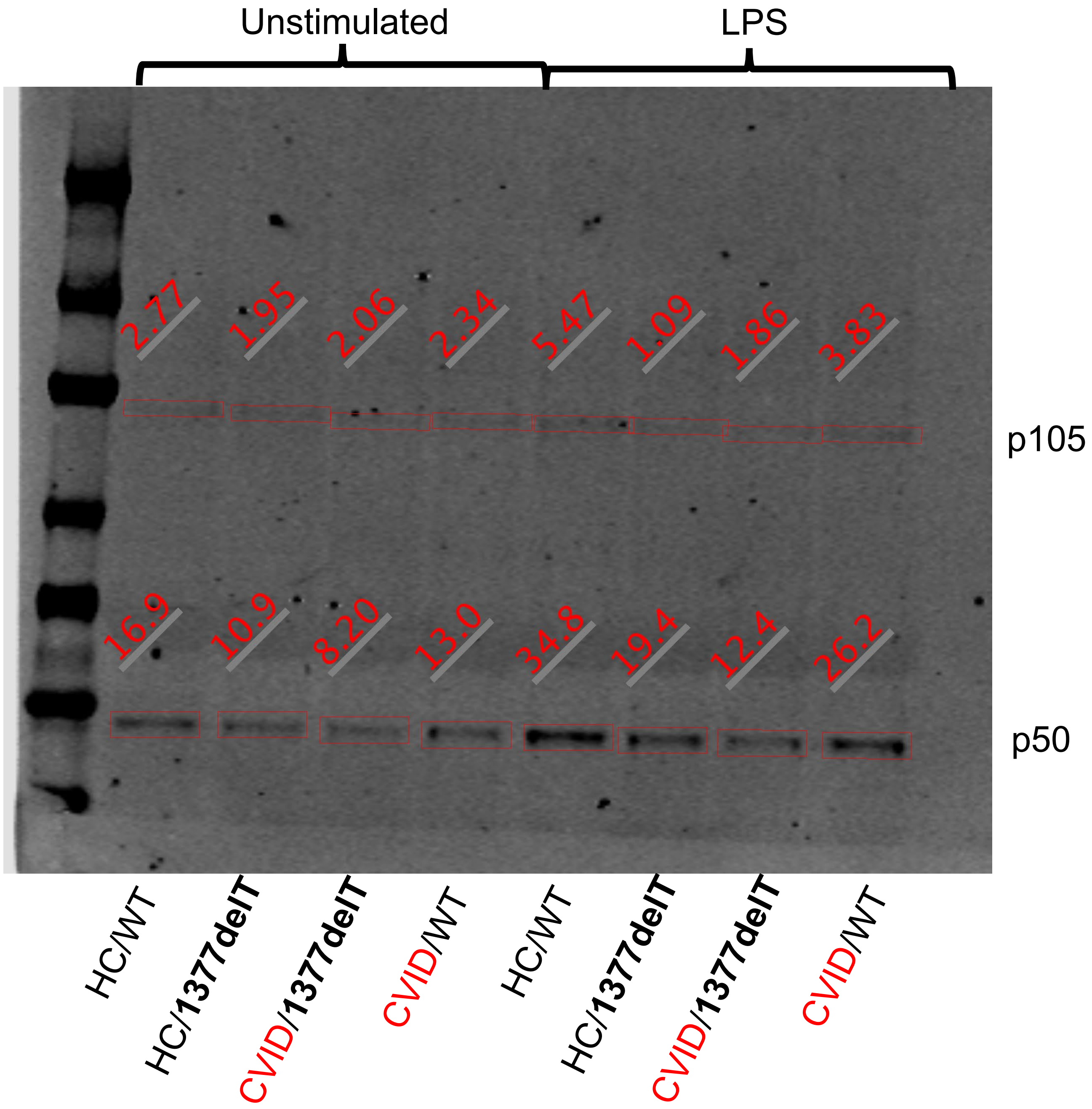

Data for Figure 4C. p50/p105 Replicate Blot 2 (Including densitometry boxes and signal values)

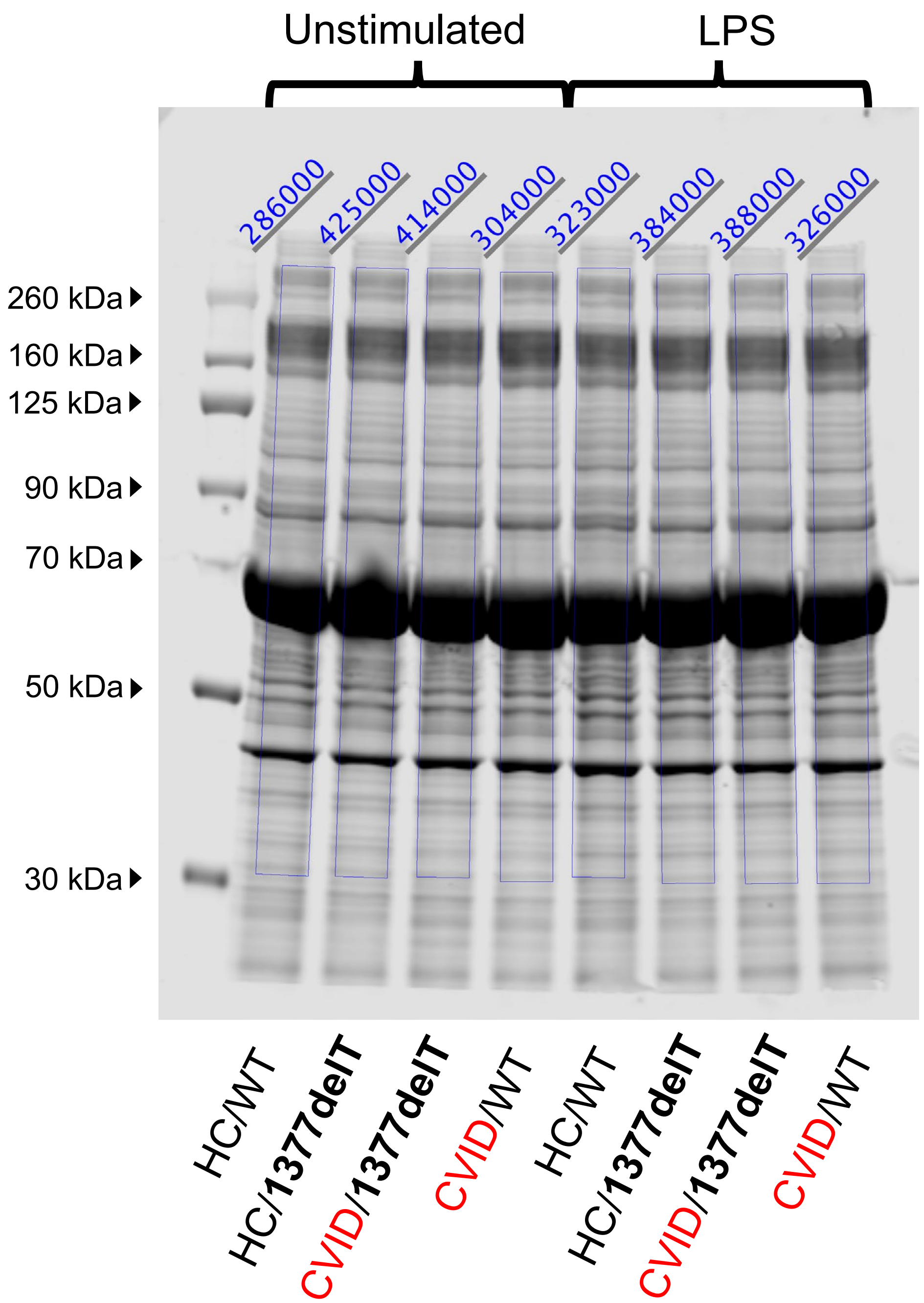

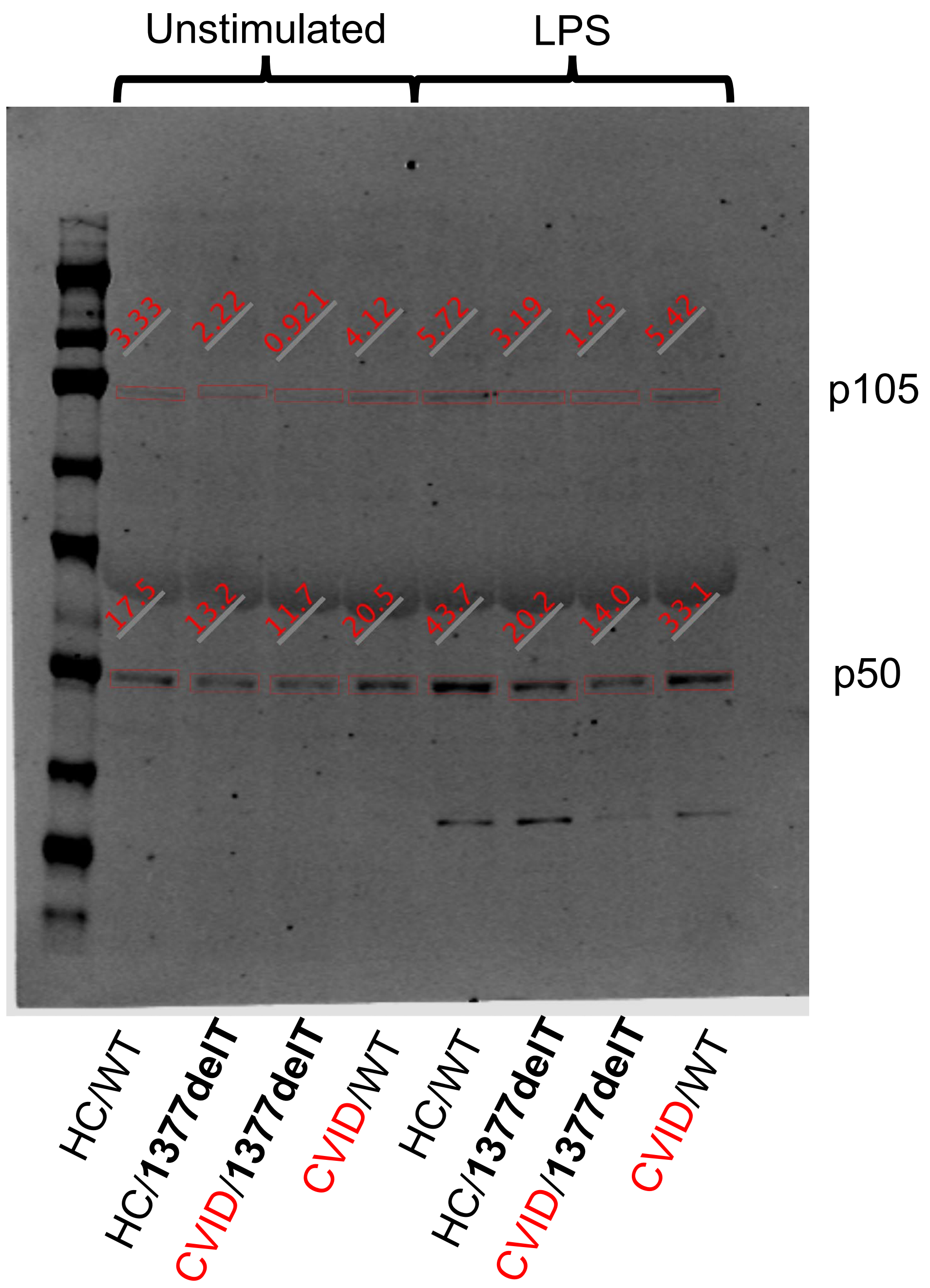

Data for Figure 4C. p50/p105 Replicate Blot 3 (Including densitometry boxes and signal values)

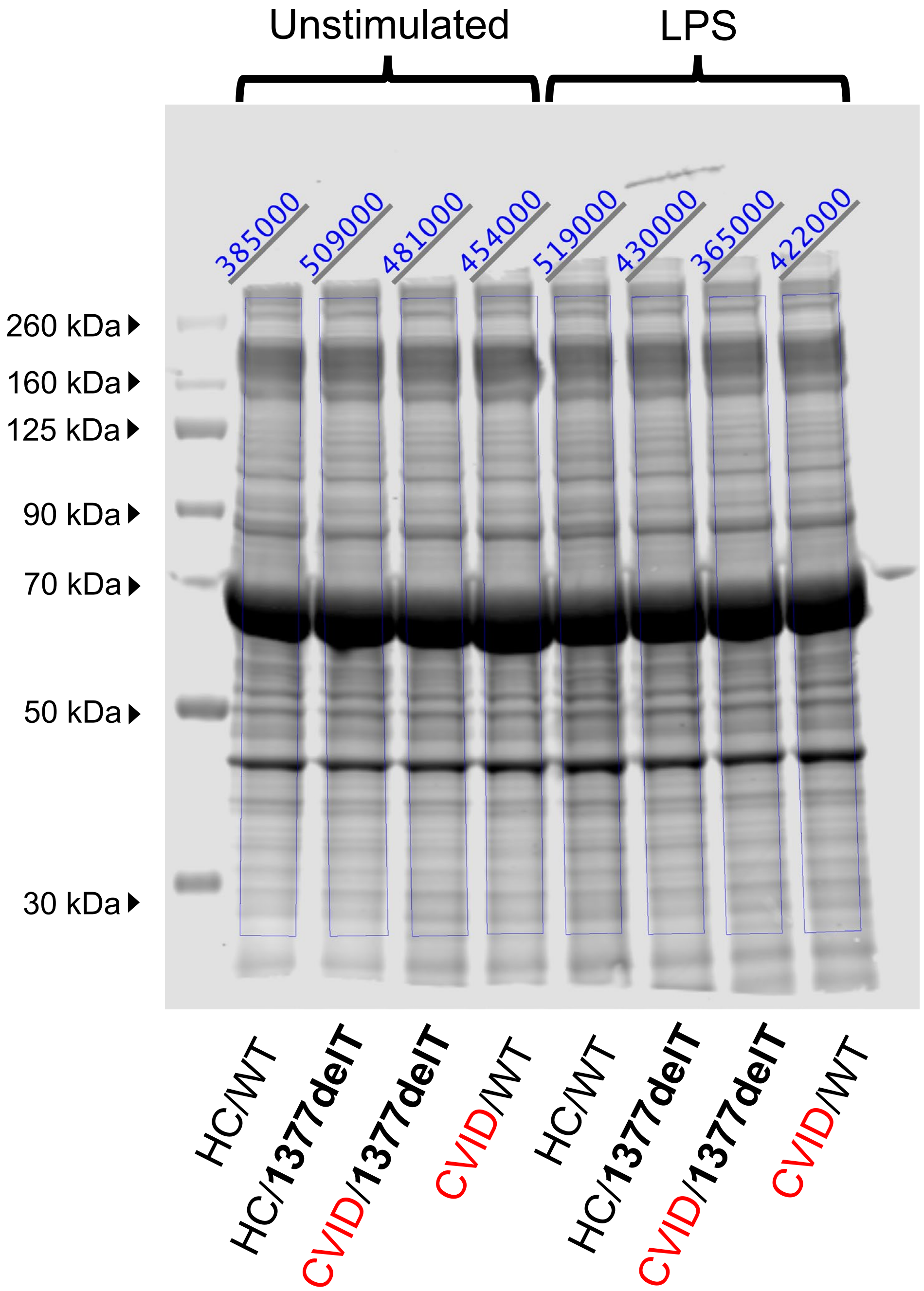

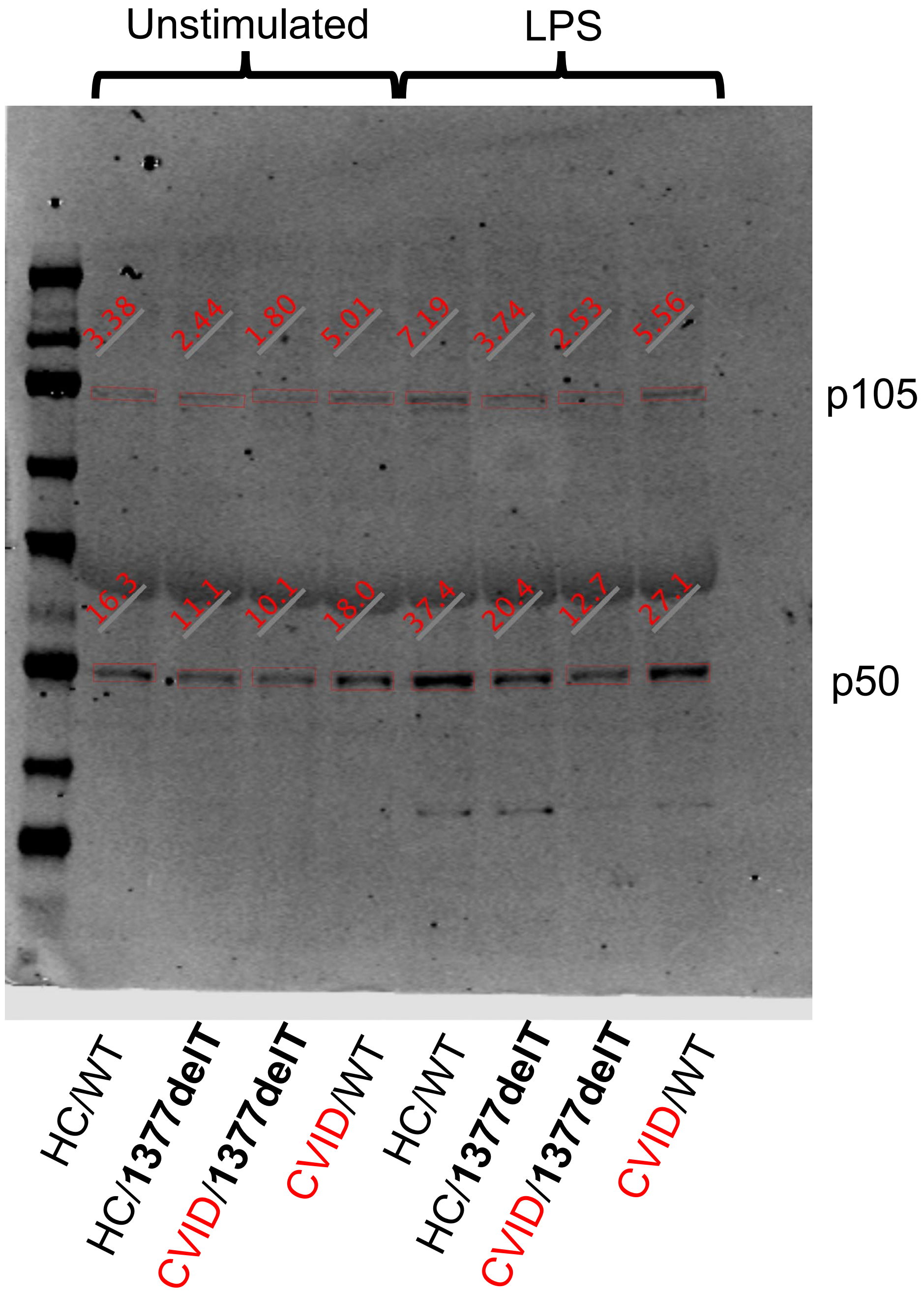

Supplementary Figure 3A Data
p105

PBMC
cytosolic fraction

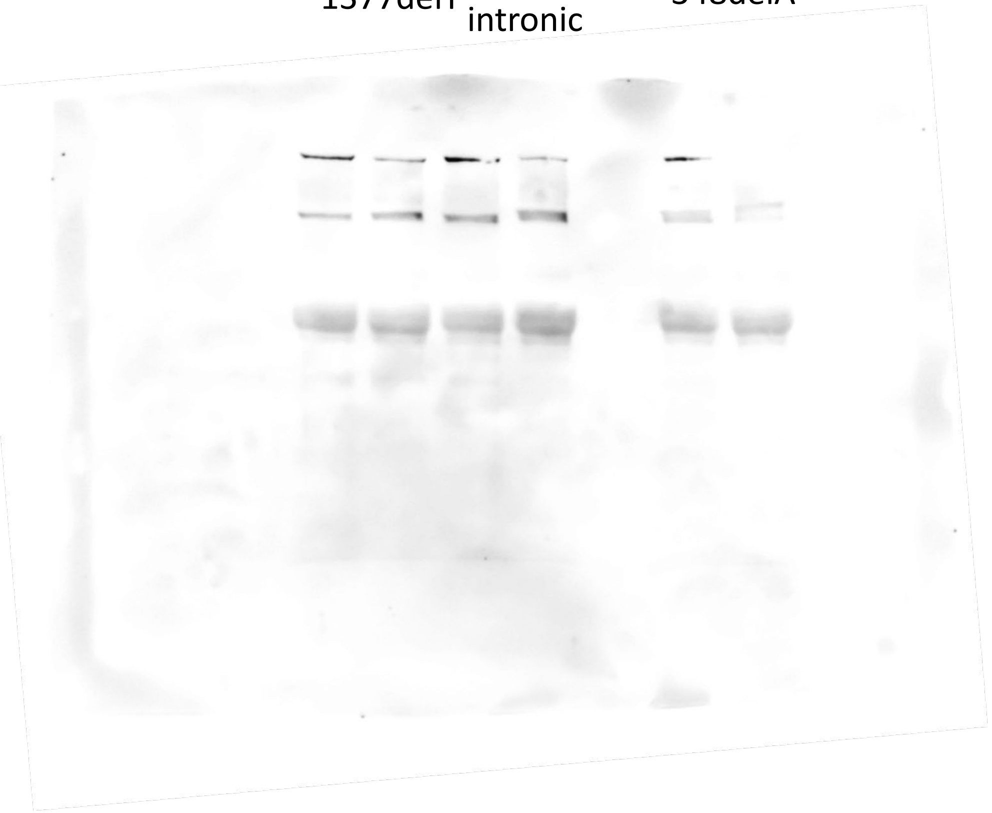

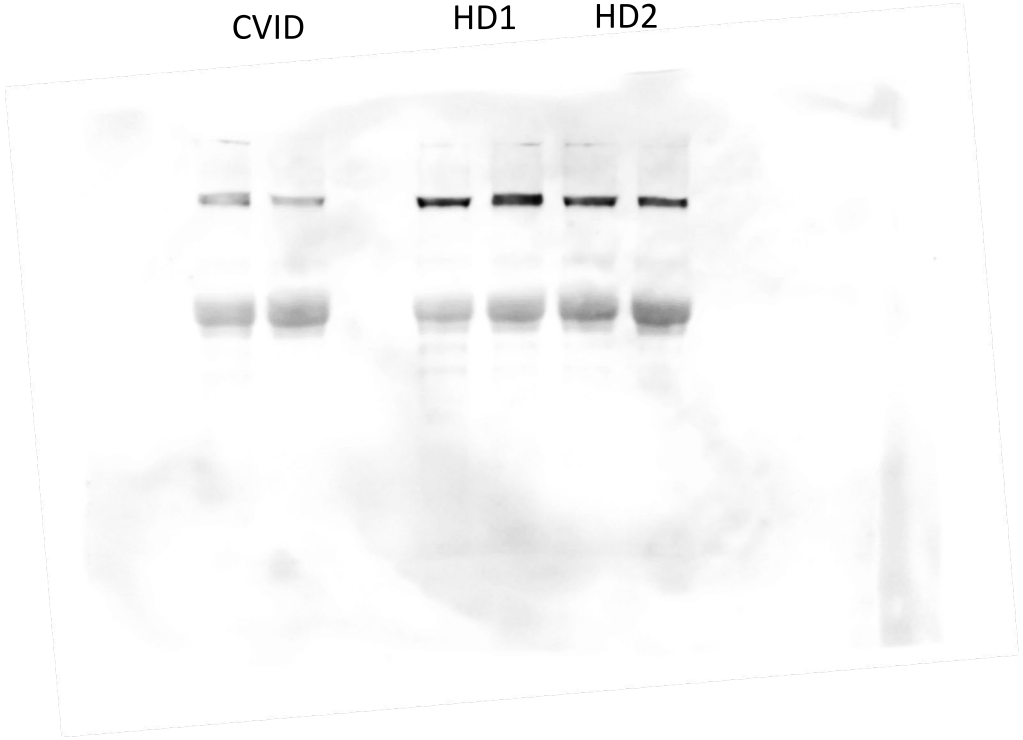

Supplementary Figure 3A Data
Phospho-p105

PBMC
cytosolic fraction

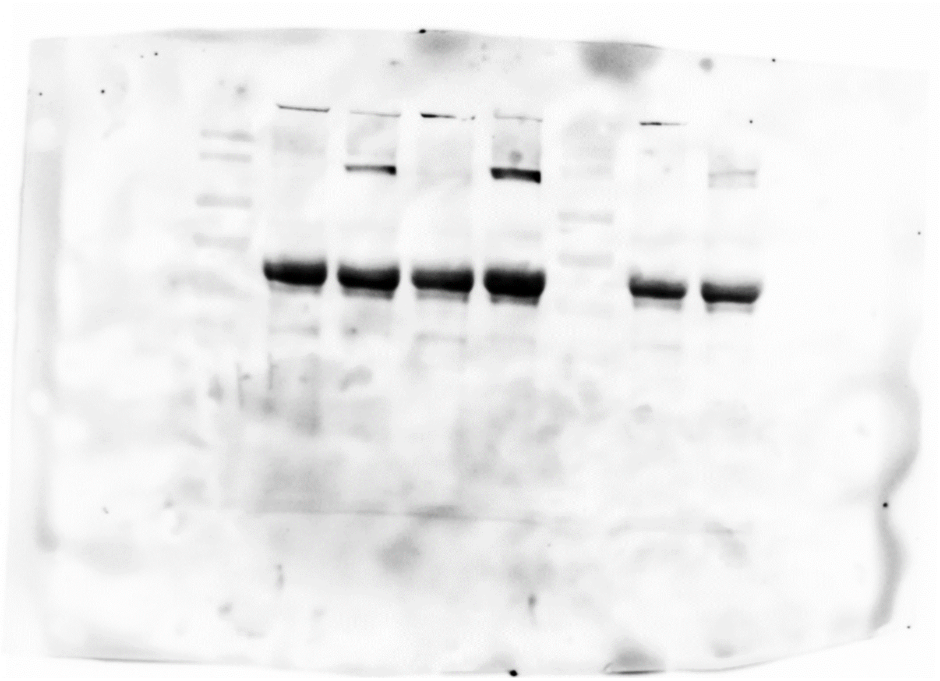

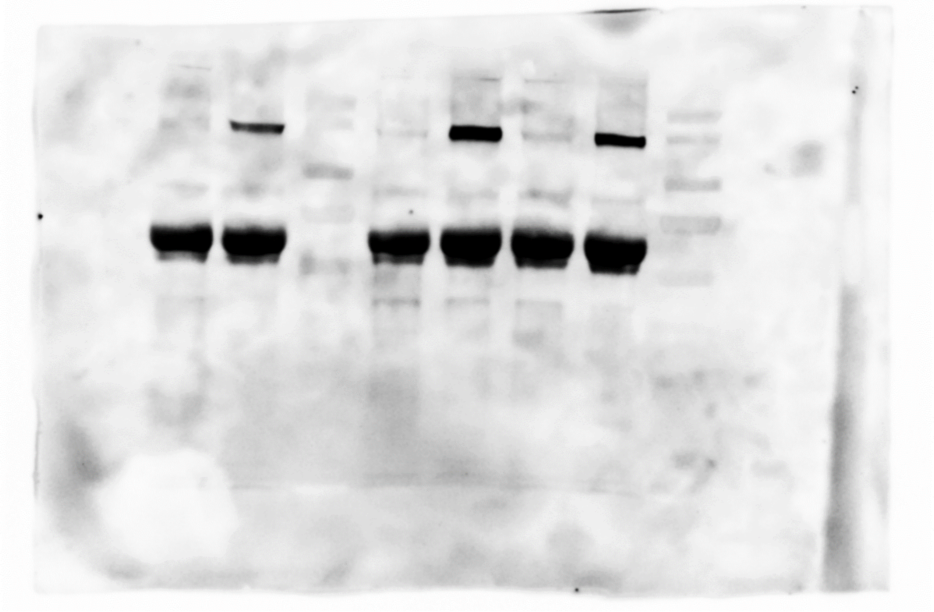

Supplementary Figure 3A Data
GAPDH

PBMC
cytosolic fraction

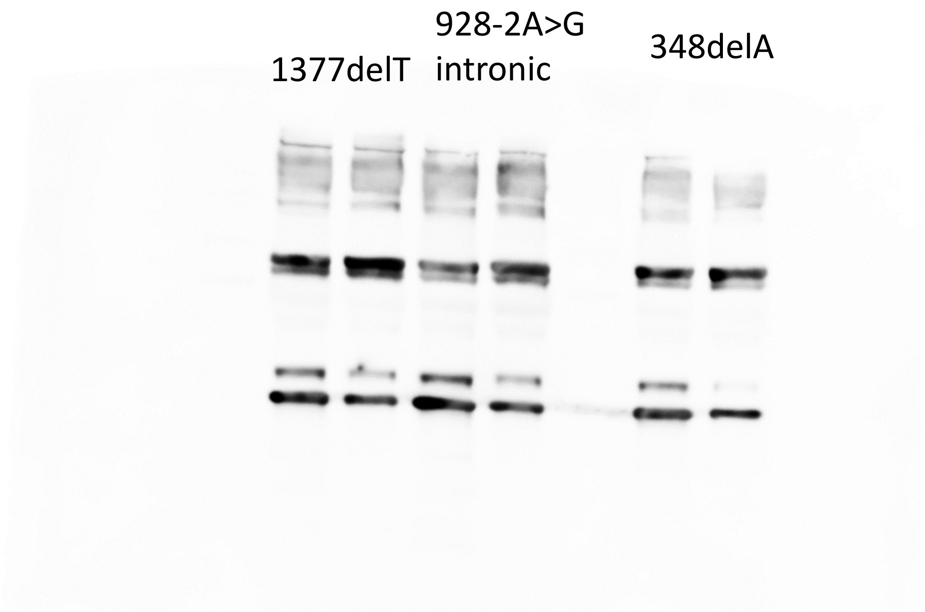

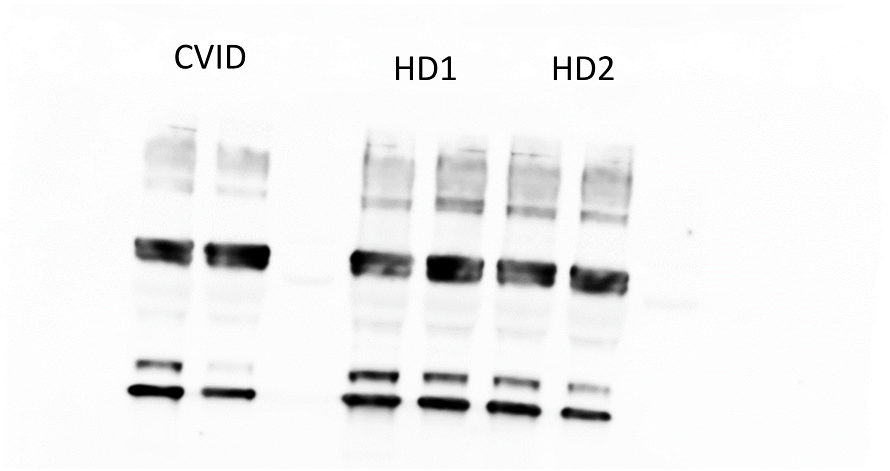

Supplementary Figure 3A Data
p50 (p105/p50)

PBMC
nuclear fraction

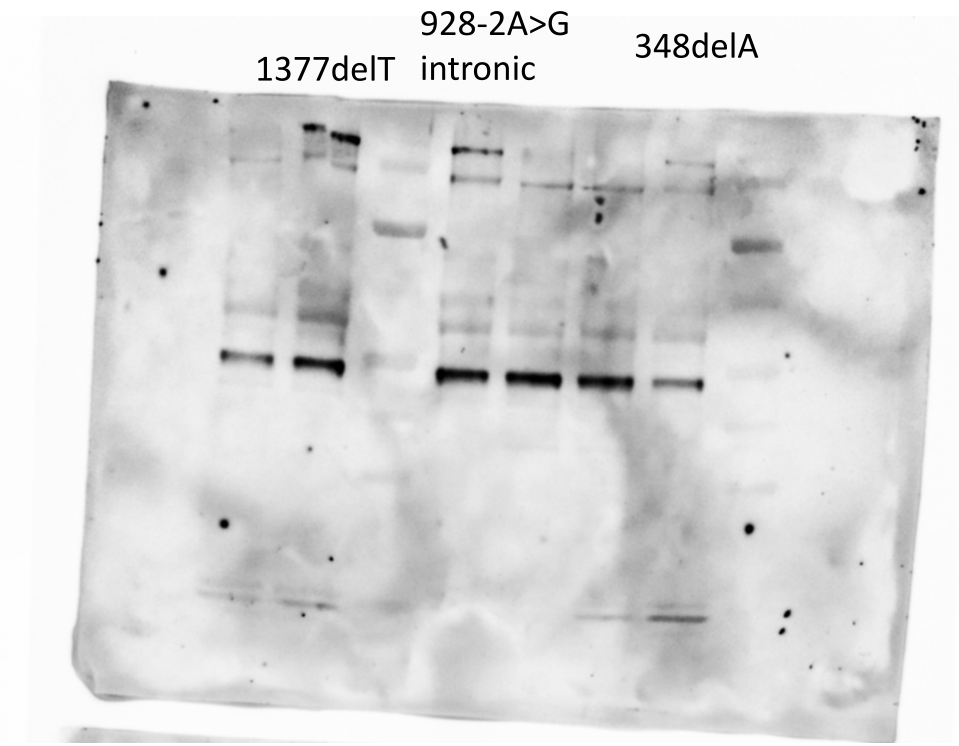

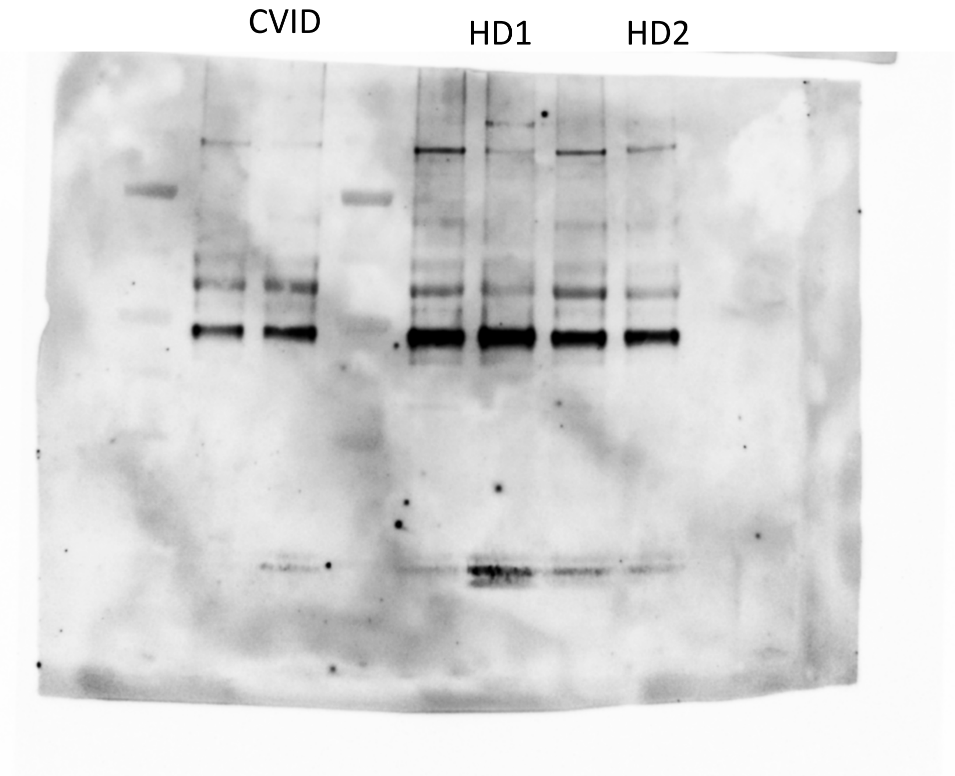

Supplementary Figure 3A Data

GAPDH

PBMC

nuclear fraction

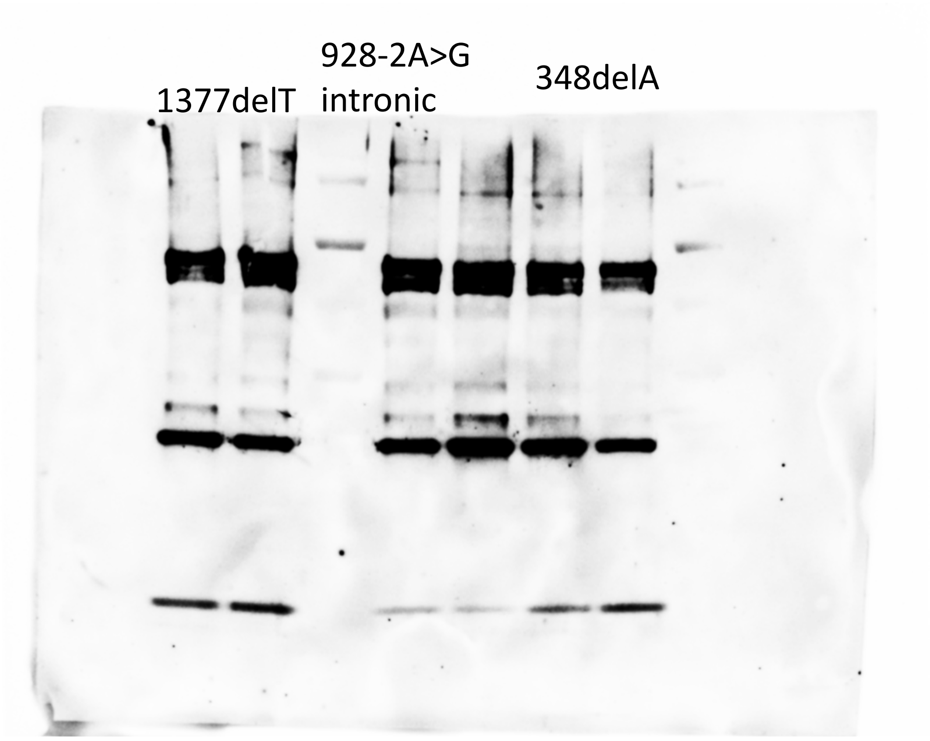

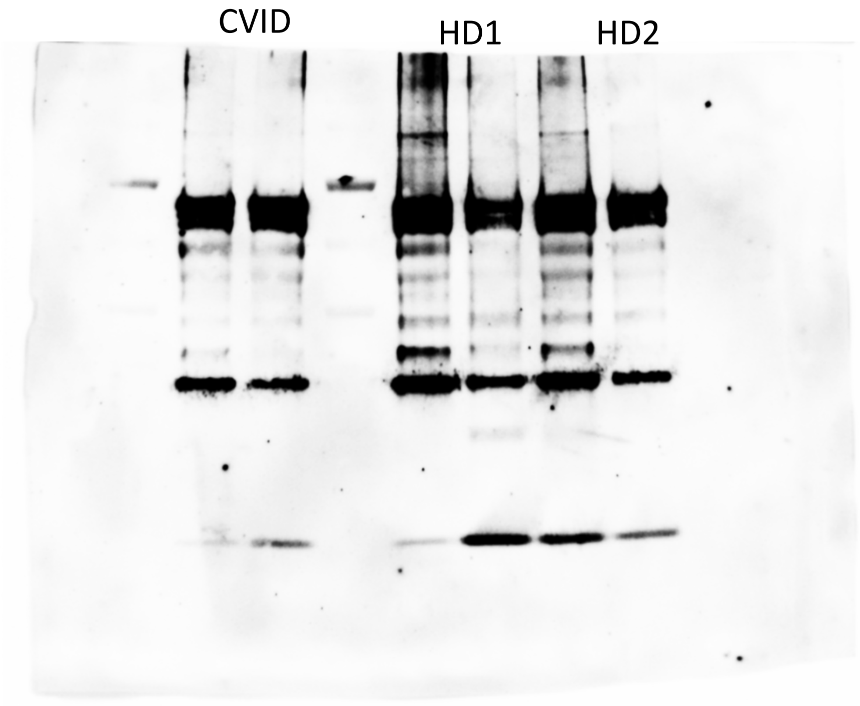

Supplement: Unedited blot and gel images [file jciinsight-11-198703-s048.pdf]
